# Thermodynamic and electrochemical study of tailor-made crown ethers for redox-switchable (pseudo)rotaxanes

**DOI:** 10.3762/bjoc.16.209

**Published:** 2020-10-20

**Authors:** Henrik Hupatz, Marius Gaedke, Hendrik V Schröder, Julia Beerhues, Arto Valkonen, Fabian Klautzsch, Sebastian Müller, Felix Witte, Kari Rissanen, Biprajit Sarkar, Christoph A Schalley

**Affiliations:** 1 Institut für Chemie und Biochemie, Freie Universität Berlin, Arnimallee 20, 14195 Berlin, Germany; 2 present address: Department of Chemical and Biological Engineering, Princeton University, Princeton, NJ08544, USA; 3 Institut für Chemie und Biochemie, Freie Universität Berlin, Fabeckstr. 34/36, 14195 Berlin, Germany; 4 present address: Lehrstuhl für Anorganische Koordinationschemie, Institut für Anorganische Chemie, Universität Stuttgart, Pfaffenwaldring 55, 70569 Stuttgart, Germany; 5 Department of Chemistry, University of Jyvaskyla P. O. Box 35, 40014 Jyväskylä, Finland

**Keywords:** crown ether, isothermal titration calorimetry, redox chemistry, rotaxanes, supramolecular chemistry

## Abstract

Crown ethers are common building blocks in supramolecular chemistry and are frequently applied as cation sensors or as subunits in synthetic molecular machines. Developing switchable and specifically designed crown ethers enables the implementation of function into molecular assemblies. Seven tailor-made redox-active crown ethers incorporating tetrathiafulvalene (TTF) or naphthalene diimide (NDI) as redox-switchable building blocks are described with regard to their potential to form redox-switchable rotaxanes. A combination of isothermal titration calorimetry and voltammetric techniques reveals correlations between the binding energies and redox-switching properties of the corresponding pseudorotaxanes with secondary ammonium ions. For two different weakly coordinating anions, a surprising relation between the enthalpic and entropic binding contributions of the pseudorotaxanes was discovered. These findings were applied to the synthesis of an NDI-[2]rotaxane, which retains similar spectroelectrochemical properties compared to the corresponding free macrocycle. The detailed understanding of the thermodynamic and electrochemical properties of the tailor-made crown ethers lays the foundation for the construction of new types of molecular redox switches with emergent properties.

## Introduction

Pedersen discovered crown ethers in 1967 while searching for multidentate ligands for the vanadyl group [1–3]. He was later awarded the Nobel Prize in Chemistry for his studies on the crown ether selective binding properties towards alkali metal ions [2]. Crown ethers and their binding properties nowadays find frequent application, e.g., as cation sensors [4–7], as phase-transfer catalysts [8–10], or as drug delivery systems [11–13].

Already at the early stages of crown ether research, considerable effort has been made towards switchable macrocyclic receptors, in which crown ethers are functionalized with a stimuli-responsive unit [14–15]. These studies were mainly motivated by a biomimetic approach and included examples such as crown ethers incorporating photo-responsive azobenzene [15–16] or redox-active ferrocene [14,17]. Yet, switchable crown ethers are also widely applied as cation sensors, where the sensor activity can be controlled by external stimuli, e.g., light, the redox potential or chemical reagents [14,17]. Redox-switchable crown ethers have been shown to sense cations by the generation of an electrochemical output. For example, crown ethers containing tetrathiafulvalene (TTF) derivatives, which enable two reversible oxidation processes from the neutral to the dicationic state, were applied to sense various cations, e.g., alkali metal ions, Pb^2+^, and Ba^2+^ [18–21].

**Figure 1:**
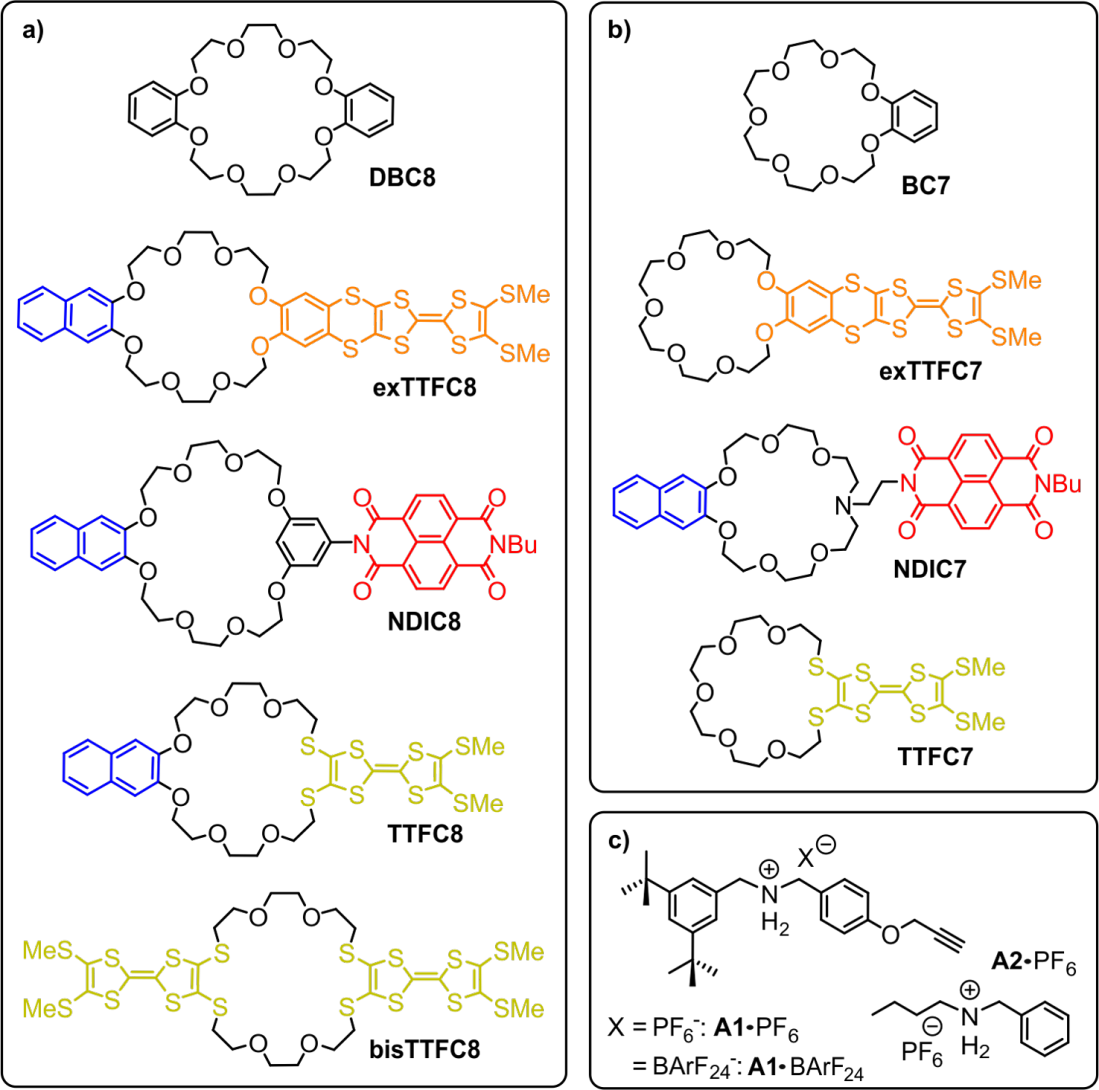
Structures of the compounds used in this study: a) crown-8 analogs; b) crown-7 analogs; c) secondary ammonium axles. BArF_24_^−^ represents tetrakis(3,5-bis(trifluoromethyl)phenyl)borate.

Furthermore, with the first synthesis of crown ether-based rotaxanes in 1995, crown ethers played a crucial role in the development of mechanically interlocked molecules (MIMs) [22–23]. This rotaxane synthesis was facilitated by the formation of a threaded complex (pseudorotaxane) between a secondary ammonium ion and dibenzo-24-crown-8 (**DBC8**, Figure 1), which bind through noncovalent interactions. In detail, these interactions are strong hydrogen bonds between ether oxygen atoms and ammonium protons. In addition, weaker C–H···O hydrogen bonds with the CH_2_ groups adjacent to the ammonium nitrogen as well as π–π-interactions between the catechol ring of the crown ether and aromatic moieties of the secondary ammonium ion contribute to the complex formation [22–25].

Over the last 25 years, a detailed understanding of the thermodynamic and kinetic properties of crown ether/ammonium complexes has developed enabling the construction of more complex molecular structures [24,26–27]. With the introduction of stimuli-responsive units, crown ether/ammonium-based MIMs have evolved into molecular switches and motors [24,28]. Intriguing examples among them are a light-powered molecular pump [29], a chemical-fuel-driven molecular rotary motor [30], and an acid/base-switchable asymmetric organocatalyst [31].

In addition to MIMs switchable by light or chemical reagents, redox-switchable molecular assemblies are of particular interest, since redox switching at electrodes is considered to operate without chemical waste and electrochemical analytical tools, e.g., cyclic voltammetry (CV), exhibit great potential to investigate the kinetic and thermodynamic parameters of the switching processes [32–33]. Although various redox-active crown ethers have been described [14,21,34], they are not commonly implemented into crown ether/ammonium (pseudo)rotaxanes. One reason is that functionalized crown ethers can cause major obstacles in the synthesis of (pseudo)rotaxanes, as their functionalization can strongly interfere with the binding properties of the crown ether [24–25]. Recently, we have investigated examples for redox-switchable MIMs based on two 24-crown-8 ethers functionalized with TTF **TTFC8** [35–36] and **exTTFC8** [37] (Figure 1). Several **TTFC8**-derived molecular assemblies have been studied and provided access to new switching modes [35–36,38] and emergent optoelectronic properties [35–36,39–40], demonstrating the great potential of tailor-made redox-active crown ethers for the development of new molecular switches.

Yet, a careful design of tailor-made redox-active crown ethers is of great importance for tuning the crown ether binding and redox properties to achieve the desired molecular structure and switching mode, which motivated us to conduct the present study on the thermodynamic and electrochemical properties of seven redox-active crown ethers of different ring sizes in comparison to the unfunctionalized analogs **DBC8** and **BC7** (Figure 1). Crown ethers incorporating TTF, an extended TTF, and naphthalene diimide (NDI) as redox-active units were investigated with respect to the impact of the functionalization on the thermodynamic binding properties towards secondary ammonium axles using isothermal titration calorimetry (ITC). The electrochemical switching properties of the redox-active crown ethers were examined using differential pulse voltammetry (DPV) and compared to those of their corresponding pseudo[2]rotaxanes. Additionally, we report the synthesis of a novel NDI-[2]rotaxane and study the impact of the mechanical bond on the optoelectronic properties of the NDI unit by CV and spectroelectrochemical measurements.

## Results and Discussion

### Design considerations

The nine crown ether wheels and two ammonium axles used in this study are depicted in Figure 1. Previously, we investigated the thermodynamic and electrochemical properties of pseudorotaxanes made from **TTFC8** and **exTTFC8** in two separate studies [35,37]. Herein, we compare these two crown ethers, their smaller analogs and add a two TTF-units containing crown ether **bisTTFC8**, which was previously synthesized by Becher and co-workers [41]. These TTF-containing crown ethers become positively charged upon electrochemical oxidation, resulting in Coulomb repulsion with the ammonium axle [35–36,38].

A second goal was to add crown ethers, that can be reversibly reduced from the neutral to the dianionic state, as these crown ethers become negatively charged upon electrochemical switching and thus are expected to cause a Coulomb attraction between the ammonium axle and the crown ether. The NDI moiety, which is readily applied in various redox-active MIMs, was selected because of its high stability and synthetic accessibility [42].

An NDI-containing crown ether was reported by Sanders and co-workers, where the NDI unit is directly connected into the oligoglycol ring framework of the crown ether by the two nitrogen atoms [43]. However, the increased ring size and altered binding properties, which are dominated by the large π-systems of the NDI units, rendered this design unsuitable for our study. Therefore, we chose a similar design for the targeted NDI-functionalized crown ether as for the TTF crown ethers, where the NDI unit is in a position more remote from the crown ether binding site. Yet, keeping the formal *C*_2_-symmetry of the macrocycle is important to avoid mixtures of isomers upon the threading of directional axles, such as **A1·**PF_6_ (Figure 1c) [40]. Consequently, we chose to use a resorcinol-connected crown ether motif introduced by Stoddart and co-workers [44] for the NDI-crown-8 **NDIC8** (Figure 1).

To compare the larger crown ethers with smaller analogs, benzo-21-crown-7 (**BC7**) and the derivatives were also included, as at least the parent compound forms stronger complexes with secondary ammonium ions than the larger analog. But since phenyl groups already act as a stopper for **BC7**, one side of the ammonium axle must be an alkyl chain, as in **A2·**PF_6_ to enable pseudo[2]rotaxane formation (Figure 1c) [45–46].

The smaller TTF-containing crown ethers **TTFC7** and **exTTFC7** were designed in analogy to their crown-8 analogs (Figure 1). To access the NDI-functionalized crown-7 analog **NDIC7**, we chose an aza-crown-7 core, similar to the divalent crown ether described by Das and co-workers [47]. Comparing both NDI-containing crown ethers, **NDIC8** exhibits a rather rigid connection to the crown ether core, and in **NDIC7**, a more flexible ethylenediamine linker is used.

### Crown ether synthesis and crystal structures

**Scheme 1:**
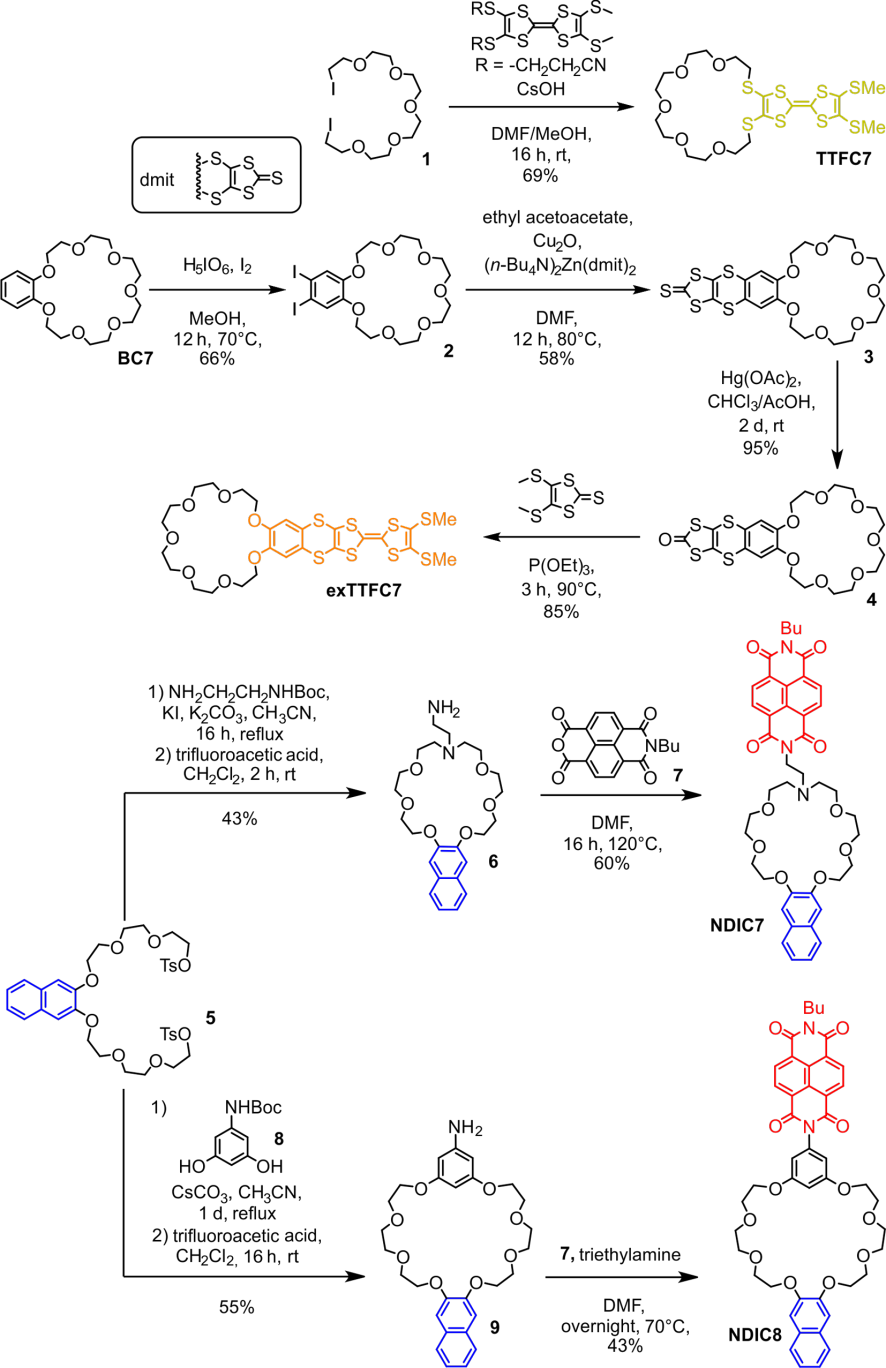
Schematic representation of synthetic routes towards **TTFC7**, **exTTFC7**, **NDIC7**, and **NDIC8**.

With respect to the synthesis of previously reported **TTFC8** and **exTTFC8** [35,37], we synthesized the novel 21-crown-7 analogs following a similar synthetic route, yielding **TTFC7** in one step and with good yield of 69% from the diiodide **1**, and **exTTFC7** with a good yield (31% over 4 steps) from **BC7** (Scheme 1, for the detailed synthetic procedure and characterization data, see Supporting Information File 1, section 1). Both NDI macrocycles **NDIC8** and **NDIC7** were synthesized in moderate yields of 24% and 26%, respectively, over three steps from the same two building blocks, the ditosylate **5** and the monobutyl-protected NDI precursor **7** (Scheme 1).

**Figure 2:**
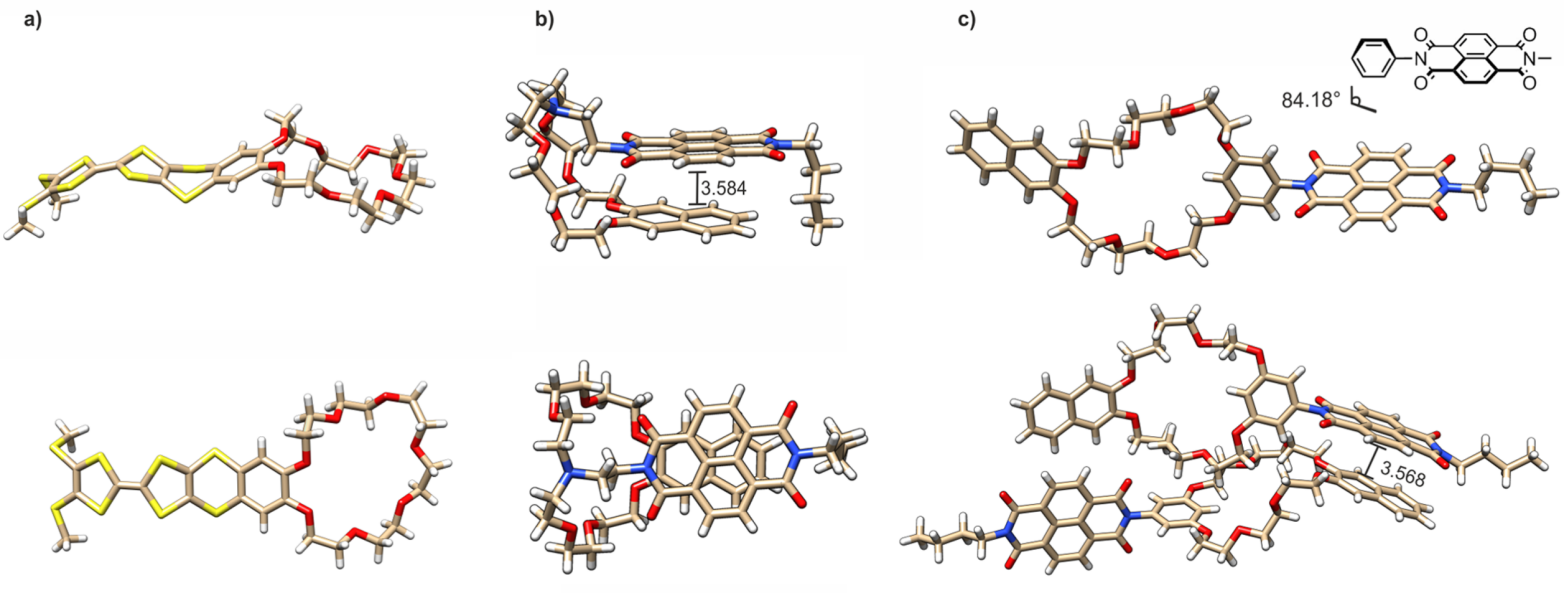
Solid-state structures of a) **exTTFC7** (CH_3_CN molecule omitted for clarity), b) **NDIC7** (CH_3_CN molecule omitted for clarity) and c) **NDIC8**.

The connectivity and conformation of **exTTFC7** was observed in the crystal structure obtained from crystals generated through slow evaporation of a CH_2_Cl_2_/CH_3_CN solution. The structure of the exTTF unit does not exhibit any significant changes upon incorporation into the crown ether [48]. No intermolecular stacking between the exTTF units was observed in the crystal structure of **exTTFC7** (Figure 2a and section 2 in Supporting Information File 1). Slow diffusion of CH_3_CN into a concentrated solution of **NDIC7** in CH_2_Cl_2_ yielded single crystals suitable for X-ray diffraction (Figure 2b). The macrocycle displays a folded conformation in the solid state due to the flexible linker, featuring an intramolecular NDI/naphthalene stacking with a typical π-stacking distance of 3.58 Å and a tilt angle of 5.8°. The free electron pair of the tertiary amine points towards the inside of the crown ether. In contrast, single crystals of **NDIC8** (Figure 2c), obtained by slow evaporation of a concentrated dimethylformamide (DMF) solution, exhibit a non-folded conformation. The torsional angle between the central phenyl ring and the NDI is 84.2° in order to avoid strain between the protons of the resorcinol and the carbonyl groups of the NDI. Consequently, an intramolecular π–π-interaction with the naphthalene on the other side of the macrocycle is impeded. **NDIC8** therefore does not fold but stacks with the naphthalene and NDI moieties of the neighbors alternatingly in the solid state and with a typical plane/plane distance of 3.57 Å [49].

### Thermodynamic analysis of crown ether/ammonium complexes

For the investigation of the thermodynamic binding properties, isothermal titration calorimetry is an advantageous method as it yields the binding stoichiometry, the binding constant *K*_a_ and the binding enthalpy Δ*H*^0^ in one measurement. From these data, the Gibbs free binding energy Δ*G*^0^ and the binding entropy Δ*S*^0^ can be calculated. Pseudo[2]rotaxanes formed from crown ethers and ammonium axles are generally more strongly bound in solvents with low dielectric constants and in combination with weakly coordinating anions (WCAs) [24,50–51]. Therefore, we chose 1,2-dichloroethane (DCE) as the solvent and hexafluorophosphate (PF_6_^−^) as the counter ion, which resulted in binding constants in the optimal range for ITC titrations [52–53]. The even more weakly coordinating tetrakis(3,5-bis(trifluoromethyl)phenyl)borate (BArF_24_^−^) anion exemplarily served for comparison to study the influence of the anion on the binding constant.

Two different ammonium axles were employed: On the one hand, **A1·**PF_6_ can only form a threaded complex with crown-8 ether derivatives due to the two bulky benzyl substituents on the ammonium ion. On the other hand, **A2·**PF_6_ possesses one narrow alkyl substituent allowing both crown-8 and crown-7 ethers to form threaded complexes.

All possible pseudo[2]rotaxanes show strongly enthalpy-driven binding – with the notable exception of **NDIC7** (Table 1). The unfunctionalized crown ethers **BC7** and **DBC8** are the strongest binders among the corresponding derivatives with Δ*G*^0^ of −34.6 kJ/mol and −34.8 kJ/mol, respectively (Table 1, entries 1 and 9).

**Table 1:** Thermodynamic binding data of different crown ether/secondary ammonium axle complexes obtained by ITC titrations in DCE at 298 K (for full data set and titration curves, see Supporting Information File 1, section 3).

entry	macrocycle	axle	*K*_a_ [10^3^ M^−1^]	Δ*G*^0^ [kJ/mol]	Δ*H*^0^ [kJ/mol]	*T*Δ*S*^0^ [kJ/mol]

1	**BC7**	**A2·**PF_6_	1200 ± 100	−34.6 ± 0.2	−63.0 ± 0.5	−28.3 ± 0.7
2	**exTTFC7**		260 ± 30	−30.9 ± 0.3	−63.6 ± 1.0	−32.7 ± 1.3
3	**NDIC7**^a^		n. d.	n. d.	n. d.	n. d.
4	**TTFC7**		8.0 ± 1.0	−22.3 ± 0.2	−55.8 ± 1.5	−33.4 ± 1.7

5	**DBC8**	**A2·**PF_6_	480 ± 70	−32.4 ± 0.3	−60.4 ± 1.5	−28.0 ± 1.8
6	**exTTFC8**		160 ± 20	−29.7 ± 0.3	−57.2 ± 2.0	−27.5 ± 2.3
7	**NDIC8**		13 ± 1	−23.4 ± 0.2	−48.1 ± 1.0	−24.7 ± 1.2
8	**TTFC8**		7.0 ± 1.0	−22.1 ± 0.2	−50.3 ± 1.0	−28.3 ± 1.2

9	**DBC8**	**A1·**PF_6_	1300 ± 100	−34.8 ± 0.3	−60.9 ± 2.0	−26.1 ± 2.3
10	**exTTFC8**		780 ± 70	−33.6 ± 0.2	−58.6 ± 0.9	−25.0 ± 1.1
11	**NDIC8**		49 ± 6	−26.7 ± 0.3	−46.6 ± 2.0	−19.9 ± 2.3
12	**TTFC8**		33 ± 3	−25.7 ± 0.2	−51.5 ± 0.9	−25.9 ± 1.1

13	**NDIC8**	**A1·**BArF_24_	1000 ± 100	−34.2 ± 0.2	−42.9 ± 1.2	−8.7 ± 1.4
14	**TTFC8**^b^		440 ± 100	−32.2 ± 0.3	−46.2 ± 0.7	−14.0 ± 1.0
15	**bisTTFC8**		2.0 ± 0.5	−18.7 ± 0.6	−21.0 ± 2.0	−2.2 ± 2.6

^a^ITC titrations cannot be fitted to a 1:1 pseudo[2]rotaxane binding model (for details, see text below and Supporting Information File 1, Figure S5). ^b^Taken from a previous report [40].

Focusing on the combinations of crown-8 ethers and **A1·**PF_6_, **exTTFC8** also forms a strong complex, with Δ*G*^0^ being only ≈1 kJ/mol (Table 1, entry 10) lower than that of the **A1·**PF_6_@**DBC8** complex. In contrast, the binding of **TTFC8** is more than 9 kJ/mol weaker (Table 1, entry 12), caused by the weaker hydrogen-bond-acceptor ability of the sulfur atoms incorporated in the **TTFC8** crown ether ring [34,54]. **NDIC8** also exhibits a comparably low binding energy, likely due to the increased ring size and the consequently weakened hydrogen-bonding pattern (Table 1, entry 11) [44].

However, **NDIC8** and **TTFC8** differ significantly in the entropic and enthalpic contributions to the binding energy (Table 1, entries 11 and 12). The comparably rigid structure of **NDIC8** is unable to adjust the conformation in the complex to achieve an optimal hydrogen-bonding pattern with the ammonium axle, and simultaneously, maximized π–π-interactions with the axle because of the 84° torsional angle between the resorcinol and the NDI unit. Consequently, the complexation of **NDIC8** is less enthalpically favored than that of **TTFC8**. However, the more rigid structure of **NDIC8** also leads to a lower degree of conformational fixation in the pseudorotaxane of **NDIC8**, and thus to a more favorable binding entropy compared to the pseudorotaxane of **TTFC8**. For **TTFC8**, the increased binding enthalpy can be explained by additional π–π-interactions between the naphthalene and TTF unit of the crown ether and the ammonium axle, resulting in a rather rigid crown ether conformation in the complex as compared to the free macrocycle. This loss of conformational flexibility rationalizes the increased entropic penalty. A similar trend is observed for the binding enthalpy and entropy of **NDIC8** and **TTFC8** with the axles **A2·**PF_6_ (Table 1, entries 7 and 8) and **A1·**BArF_24_ (Table 1, entries 13 and 14), showing that this effect is caused by the macrocycle and not the ammonium axle.

The Gibbs free binding energy Δ*G*^0^ of all four crown-8 ethers to axle **A2·**PF_6_ is collectively 2–4 kJ/mol lower (Table 1, entries 5–8) in comparison to that of **A1·**PF_6_. On the one hand, additional π–π-interactions of the phenyl ring in **A1·**PF_6_ to the crown ether aromatic rings favor the complex formation. On the other hand, the flexibility of the alkyl substituent in **A2·**PF_6_ is diminished upon complexation, inducing a larger entropic penalty visible in the overall more negative binding entropies in pseudo[2]rotaxanes of **A2·**PF_6_ as compared to those formed from **A1·**PF_6_.

Furthermore, the three crown-7 macrocycles **BC7**, **exTTFC7**, and **TTFC7** bind **A2·**PF_6_ with a binding energy (entries 1, 2, and 4 in Table 1) slightly higher than the crown-8 analogs (entries 5, 6, and 8 in Table 1). Moreover, Δ*G*^0^ follows the same trend as observed for crown-8 analogs with **A1·**PF_6_ (entries 9, 10, and 12 in Table 1) and discussed above: the binding energy decreases from **BC7** over **exTTFC7** to **TTFC7**.

The azacrown-7 **NDIC7** is an exception: The ITC titration with the ammonium axle **A2·**PF_6_ does not exhibit the anticipated sigmoidal shape of a 1:1 bonded complex (see Figure S5a in Supporting Information File 1). The curve shape suggests a more complex chemical equilibrium that involves more than one chemical process generating heat, taking place in the titration experiment. One process is likely a proton transfer from the secondary ammonium group of the axle to the tertiary amine in the crown ether wheel. An ITC titration with **A1·**PF_6_ gave a similar curve shape (see Figure S5b in Supporting Information File 1), though crown-7 ethers are too small to thread over the phenyl ring of **A1·**PF_6_ under the conditions of the experiment. The folded structure observed in the crystal structure hints towards a possible “side-on” complex, where the ammonium axle is not threading through the ring of the macrocycle, yet still forms hydrogen bonds to the crown ether [24–25] (see spectroelectrochemical measurements below). These results suggest that both ammonium axles form a similar type of equilibrium with **NDIC7**, where the protonation of the tertiary amine and the complexation in a nonthreaded complex might contribute.

When using BArF_24_^−^ as the counterion for **A1**, the binding energies increase by 6–8 kJ/mol, which results in a 10–20-fold increase of the binding constants as observed for the weaker binding macrocycles **TTFC8** and **NDIC8** (Table 1, entries 13 and 14). **A1·**BArF_24_ even allows the formation of a pseudo[2]rotaxane with the **bisTTFC8** macrocycle (Table 1, entry 15), to which **A1·**PF_6_ binds too weakly to determine the binding data by ITC. The observed decrease of the binding energy with more sulfur atoms in the crown ether ring from **DBC8** over **TTFC8** to **bisTTFC8** is consistent with a systematic study on thiacrown ethers [54].

Surprisingly, the increased Gibbs free binding energy Δ*G*^0^ for **A1·**BArF_24_ compared to **A1·**PF_6_, is not caused by the binding enthalpy Δ*H*^0^ (entries 11–14 in Table 1), as one might have expected, assuming the ion pairing to compete with the pseudorotaxane formation. In contrast, the enthalpic contribution is 4–6 kJ/mol less negative with **A1·**PF_6_ than in **A1·**BArF_24_ complexes, but the formation of the **A1·**BArF_24_ pseudo[2]rotaxanes is less entropically disfavored, reflected by 11–12 kJ/mol less negative *T*Δ*S*^0^ (Table 1, entries 13 and 14). To the best of our knowledge, the study of weakly coordinating anions in the formation of pseudorotaxane complexes has been limited to their impact on the binding constant, but enthalpic and entropic contributions have not yet been studied [50–51,55]. As PF_6_^−^ is more strongly coordinating than BArF_24_^−^, a larger fraction of **A1·**PF_6_ ion pairs is present in nonpolar solvents such as DCE. Upon complexation, the ion pair of **A1·**PF_6_ must dissociate, releasing PF_6_^−^ anions into the bulk solution where they are solvated by a number of solvent molecules. The charge-induced order of the solvent dipoles in the solvent shell is entropically unfavorable and more pronounced for PF_6_^−^. Consequently, the main reason for the observed effects is likely a change in the solvation entropy. However, to further elucidate the role of WCAs in crown/ammonium complexes, more detailed studies are certainly indicated.

### Electrochemistry

The electrochemical properties of the TTF and NDI-bearing macrocycles and pseudorotaxanes are summarized in Table 2. To get some insight into the solvent dependence of the electrochemical data, the measurements were performed in 1:1 DCE/CH_3_CN (increased solubility of the axle salts, weaker pseudo[2]rotaxane binding) and in pure DCE (stronger pseudo[2]rotaxane binding). Generally, the oxidation potentials are shifted to higher values, and the reduction potentials are shifted to lower values in pure DCE due to decreased charge stabilization.

**Table 2:** Electrochemical data obtained by differential pulse voltammetry (for voltammograms and experimental details see Supporting Information File 1, section 4).

entry	compound	solvent^a^	*E*_1/2_^red2^ [V]^b^	*E*_1/2_^red1^ [V]^b^	*E*_1/2_^ox1^ [V]^b^	*E*_1/2_^ox2^ [V]^b^
			reversible reductions		reversible oxidations	

1	**exTTFC7**	DCE/CH_3_CN 1:1	/	/	0.66	0.95
2	**exTTFC8^c^**		/	/	0.66	0.93
3	**TTFC7**		/	/	0.59	0.83
4	**TTFC8**		/	/	0.59	0.83
5	**bisTTFC8**^c^		/	/	0.57	0.93
6	**NDIC7**		−0.96	−0.54	/	/
7	**NDIC7** + **A2·**PF_6_^d^		−0.70	−0.46	/	/
8	**NDIC8**^c^		−0.95	−0.49	/	/
9	**NDIC8**^c^ + **A1·**PF_6_^d^		−0.72	−0.49	/	/
10	**NDIC8**^c^ + (CH_3_)_2_NH_2_PF_6_^d^		−0.78	−0.45		
11	**NDIC8Rot**		−0.95	−0.50		

12	**exTTFC7**	DCE	/	/	0.65	1.01
13	**exTTFC7** + **A2·**PF_6_^d^		/	/	0.67	1.01
14	**TTFC7**		/	/	0.59	0.87
15	**TTFC7** + **A2·**PF_6_^d^		/	/	0.63	0.87
16	**bisTTFC8**		/	/	0.56	0.95
17	**NDIC8**^c^		−0.97	−0.53	/	/
18	**NDIC8Rot**		−0.96	−0.51	/	/

^a^With *n*-Bu_4_NPF_6_ (0.1 M) as the electrolyte. ^b^Half-wave potentials are given against the decamethylferrocene/decamethylferrocenium couple as the reference; error = ±0.01 V. ^c^The compound showed only moderate solubility in the corresponding solvent. ^d^Five equivalents of the ammonium guest were added.

As expected, all TTF macrocycles display two reversible oxidation processes (*E*_1/2_^ox1^, TTF→TTF^•+^ and *E*_1/2_^ox2^, TTF^•+^→TTF^2+^). *E*_1/2_^ox1^ and *E*_1/2_^ox2^ of the free **exTTFC8** and **exTTFC7** crown ethers are anodically shifted compared to those of **TTFC8** and **TTFC7**, in which the TTF units are directly incorporated into the crown ethers. This behavior is known also for the two redox-active TTF building blocks **exTTF** and tetramethylene-TTF, which are not part of a macrocycle [48].

The addition of the axles **A1·**PF_6_ or **A2·**PF_6_ to **TTFC8** and **TTFC7**, respectively, in a DCE/CH_3_CN 1:1 solution has no significant effect on *E*_1/2_^ox1^ and *E*_1/2_^ox2^ (see Supporting Information File 1). However, an increase in the oxidation potential (Δ*E*_1/2_^ox1^ = +0.04 V) was observed for the **A2·**PF_6_@**TTFC7** complex in pure DCE, which can be attributed to the increased strength of the hydrogen bonds in the neutral complex and Coulomb repulsion in the oxidized complex [35]. This effect is less pronounced for the complex **A2·**PF_6_@**exTTFC7** because the TTF unit is more distant to the ammonium unit [37].

The macrocycle **bisTTFC8**, bearing two TTF units, shows a broadening of the signal for the first oxidation (Figure S9 in Supporting Information File 1), presumably due to intramolecular TTF–TTF interactions [35–36,56]. In comparison to the wheel **TTFC8**, the second oxidation of **bisTTFC8** is anodically shifted by Δ*E*_1/2_^ox2^ = +0.10 V, indicating intramolecular Coulomb repulsion between the two TTF^2+^ units. The addition of **A1·**PF_6_ does not have any impact on the redox properties of **bisTTFC8**, as hardly any pseudo[2]rotaxane forms with this axle.

The NDI-decorated wheels **NDIC8** and **NDIC7** undergo two reversible reduction processes (*E*_1/2_^red1^, NDI→NDI^•−^ and *E*_1/2_^red2^, NDI^•−^→NDI^2−^). Here, the addition of the corresponding ammonium axles **A1·**PF_6_ and **A2**−PF_6_ drastically shifts the reduction potentials in DCE/CH_3_CN 1:1 (**NDIC7**: +0.08 V and +0.26 V; **NDIC8**: 0 V and +0.23 V, for Δ*E*_1/2_^red1^ and Δ*E*_1/2_^red2^, respectively). We assume that the negatively charged reduced forms NDI^•−^ and NDI^2−^ form strong electrostatic interactions to ammonium ions and act as strong, competitive hydrogen-bond acceptors [57]. The high anodic shifts indicate strong attractive interactions between the wheels and axles, which compete with the coordination of the crown ether moiety. Thus, the secondary ammonium axles form presumably a non-threaded complex with the reduced NDI crown ethers, where the charged ions are in closer proximity than in the pseudo[2]rotaxane. This assumption was further confirmed by the addition of the hydrogen bond donor (CH_3_)_2_NH_2_PF_6_ to a solution of the wheel **NDIC8**, which again leads to strong anodic shifts in the voltammogram (Δ*E*_1/2_^red1^ = +0.04 V, Δ*E*_1/2_^red2^ = +0.17 V, Table 2, entry 10) and will be further elucidated below.

### Synthesis and spectroelectrochemical characterization of [2]rotaxane with NDI crown ether

**Figure 3:**
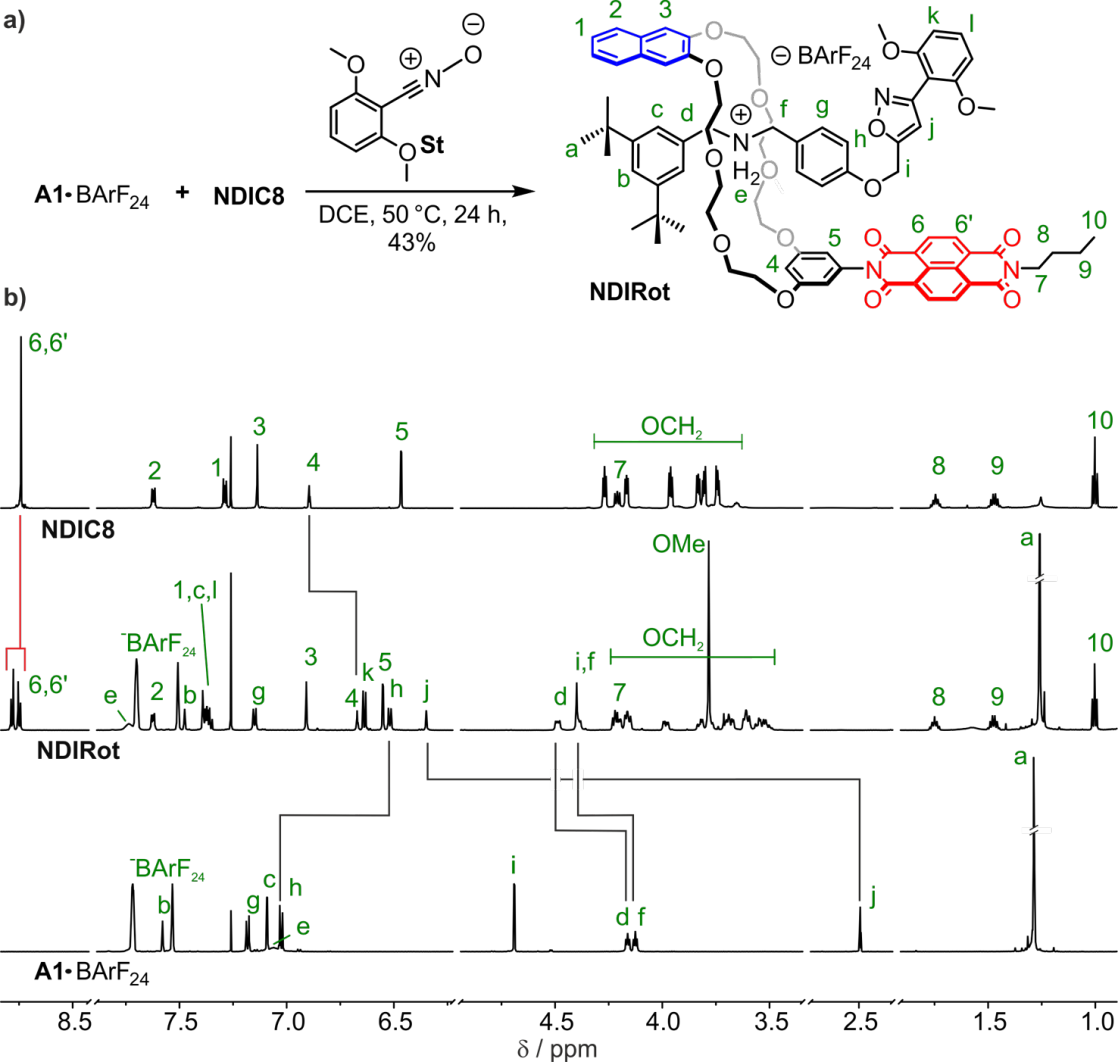
a) Synthesis of the [2]rotaxane **NDIRot**. b) Stacked ^1^H NMR spectra (700 MHz, CDCl_3_, 298 K) of **NDIC8** (top), **NDIRot** (middle), and **A1·**BArF_24_ (bottom). The signal assignment was done by 2D NMR spectroscopy.

For **TTFC8**, we have recently shown, that rotaxane formation influences the optoelectronic properties of the TTF unit [35], yet for **exTTFC8** the [2]rotaxane shows very similar properties as compared to the free macrocycle [37]. As **NDIC8** and the **A1·**PF_6_@**NDIC8** pseudo[2]rotaxane also reveal distinctly different electrochemical potentials, we investigated the impact of mechanical bonding on the optoelectronic properties of the NDI unit. The binding properties suggest that the combination of the **A1·**BArF_24_ axle and **NDIC8** is optimal for the synthesis of an NDI-containing [2]rotaxane. We applied Takata’s catalyst-free stoppering approach [58] using the nitrile oxide **St** for the preparation of the [2]rotaxane **NDIRot**, which was obtained in 43% yield (Figure 3a).

The formation of the isoxazole can be recognized by the strong downfield shift of the proton H^j^ in the ^1^H NMR spectrum (Figure 3b, for 2D spectra and signal assignment, see Supporting Information File 1, section 5.1). Furthermore, a diastereotopic splitting of the crown ether methylene protons and downfield shifts of the axle methylene protons H^d^ and H^f^ clearly point at the rotaxane formation. Additionally, a strong upfield shift observed for the resonance of the phenylic proton H^h^ (Δδ = 0.5 ppm) is in line with rotaxane formation, even though it has not previously been observed for similar TTF-containing rotaxanes [35,40]. This finding indicates a different conformation of the macrocycles in the NDI and TTF [2]rotaxanes.

Even though the signals of the NDI protons H^6^ and H^6’^ do not shift significantly which would be expected for strong π–π-interactions, they split from one pseudo singlet into two doublets upon rotaxane formation, indicating a lower symmetry of the NDI unit. The resorcinol proton H^4^ exhibits a significant upfield shift (Δδ = −0.2 ppm), which can be rationalized by the position in the crown ether cavity close to the positively charged ammonium ion.

The collision-induced dissociation of mass-selected rotaxane ions occurs only at comparably high collision energy. Only axle fragments are observed while the intact axle is not seen among the fragments (Figures S15 and S16 in Supporting Information File 1). This clearly supports the mechanically interlocked structure for **NDIRot** in analogy to similar structures investigated by tandem mass spectrometry earlier [35,40].

Cyclic voltammograms of **NDIRot** show two reversible reductions *E*_1/2_^red1^ and *E*_1/2_^red2^ independent of the used solvent (pure DCE or DCE/CH_3_CN 1:1, Figure S11 and Table S2 in Supporting Information File 1). Additionally, the two reduction potentials are very similar to those of free **NDIC8** and not the pseudo[2]rotaxane **A1·**PF_6_@**NDIC8** (see Table 2, entries 8–11). This agrees well with the assumption that the reduction of the NDI leads to a complete rearrangement of the pseudo[2]rotaxane into a non-threaded complex as discussed above. As dethreading is impossible in **NDIRot**, the electrochemical data are significantly different from those of the non-threaded complexes formed from the pseudorotaxane and the (CH_3_)_2_NH_2_PF_6_ complex of **NDIC8**.

**Figure 4:**
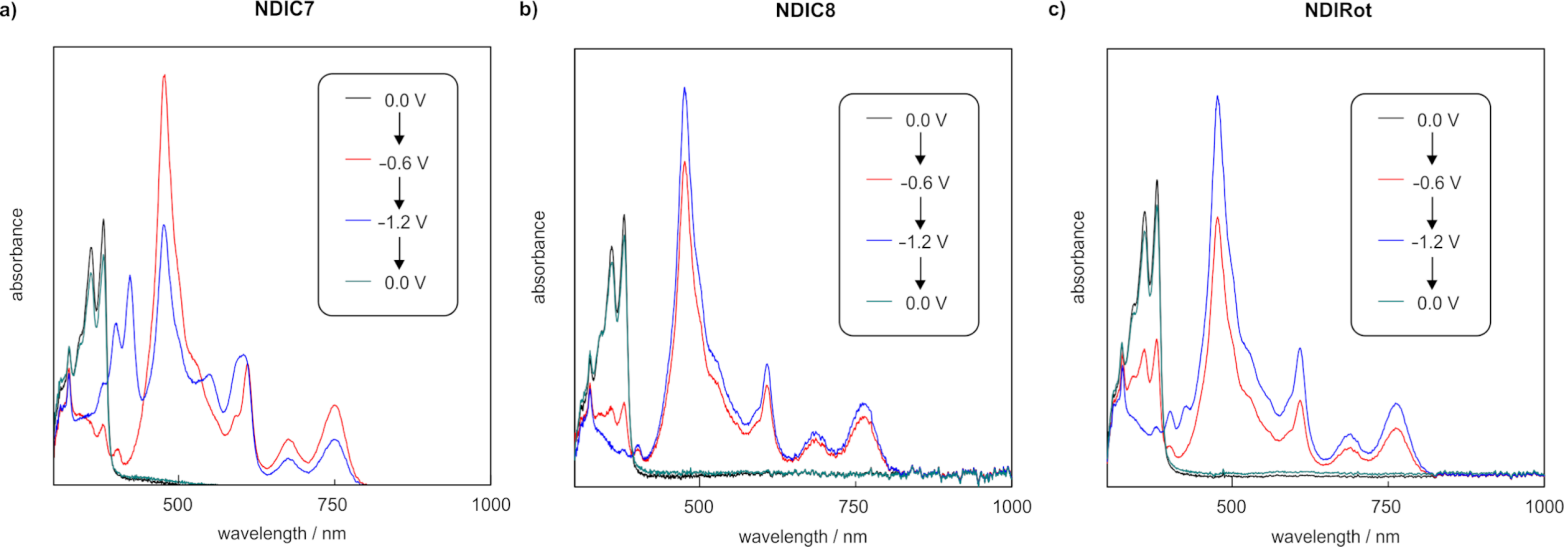
UV–vis–NIR spectra obtained by spectroelectrochemical measurements (0.1 M *n*-Bu_4_PF_6_, CH_2_Cl_2_/CH_3_CN 1:1, 298 K) of a) **NDIC7** (0.5 mM), b) **NDIC8** (0.5 mM), and c) **NDIRot** (1 mM). Potentials are referenced against a silver wire pseudo-reference electrode.

The optoelectronic properties of the rotaxane **NDIRot** were investigated by UV–vis–NIR spectroelectrochemistry in a CH_2_Cl_2_/CH_3_CN 1:1 mixture and compared to **NDIC8** and **NDIC7** (Figure 4 and Table S3 in Supporting Information File 1). In the neutral state, the rotaxane displays the typical absorption pattern of an *N*,*N’*-disubstituted NDI monomer between 300 and 380 nm (Figure 4a–c, black) [59]. A weak charge-transfer band at ≈450 nm is observed for the free macrocycle **NDIC7** (Figure 4a, black), which is most likely caused by an intramolecularly folded naphthalene–NDI complex in solution, similar to the crystal structure. In contrast, no charge-transfer band is present, neither for **NDIRot** nor for **NDIC8**, ruling out the formation of similar charge-transfer complexes in **NDIC8** or in the corresponding rotaxane.

After one-electron reduction (NDI → NDI^•−^), a complex absorption band pattern emerges in the visible region of all three spectra. These bands are indicative of the radical anion NDI^•−^ (Figure 4a–c, red curves) [59]. The radical character of **NDIC8** was additionally confirmed by spectroelectrochemical EPR measurements, which showed an isotropic signal with a g-value of 2.004 (Figure S17 in Supporting Information File 1).

Upon further reduction, a new absorption pattern emerges for **NDIC7**, in accordance to a second electrochemical reduction (NDI^•−^ → NDI^2−^) (Figure 4a, blue curve). However, only small shifts and intensity changes are observed for **NDIC8** and the rotaxane **NDIRot** when going to a more negative potential (−1.2 V), which can be explained by diffusion and comproportionation of the dianion, as it was observed earlier for other NDI^2−^ species [59]. Applying more positive potentials gradually converts the spectra back to the initial forms, which confirms the reversibility of the reduction processes. Overall, the comparison of all absorption spectra clearly demonstrates that the rotaxane formation does not significantly influence the optoelectronic properties of the NDI unit. This can be explained by the position of the NDI moiety being rather remote from the binding site of the crown ether.

## Conclusion

In conclusion, the comparison of the thermodynamic properties of a series of functionalized crown ethers showed a small effect of redox-active units remotely attached to the crown ether on the binding of secondary ammonium ions. However, if the redox-active unit is incorporated directly into the crown ether core, as in **TTFC8** and **TTFC7**, the binding properties are altered significantly. The redox properties do not depend much on the crown ether core, irrespective of whether the redox-active unit is attached in a remote position or incorporated in the crown ether.

Our findings provide some guidelines for how the binding and redox-switching properties can be fine-tuned for the construction of a desired crown ether-based switchable MIM: while **bisTTFC8** shows interesting redox properties but very low binding constants and **exTTFC8** displays a high binding constant, yet no strong interaction of the ammonium-binding site and the redox unit, **TTFC8** offers the best compromise of sufficiently high binding constants combined with sufficient Coulomb repulsion between the oxidized TTF and the ammonium ion to construct a molecular switch [35]. This trend can directly be translated to the smaller **exTTFC7** and **TTFC7**, which exhibit very similar thermodynamic and redox properties as compared to the corresponding crown-8 derivatives.

The important role that weakly coordinating counterions play in the binding of crown ether/ammonium pseudorotaxanes needs to be emphasized: a significant binding enhancement is achieved when BArF_24_ anions are used compared to hexafluorophosphate. Surprisingly, this increased binding energy is not caused by a higher binding enthalpy, but can be attributed to a favorable change in the solvation entropy.

Two novel NDI-containing crown ethers have been successfully synthesized and characterized. Both compounds exhibit two reversible reduction processes. The lariat ether **NDIC7** is not suitable for rotaxane synthesis as it forms a complex equilibrium involving the deprotonation of the secondary ammonium axle and does not form 1:1 pseudorotaxanes. Additionally, the pseudorotaxane is hampered by intramolecular folding which was observed in the solid-state structure and is likely also present in solution, as indicated by a charge-transfer band. Nevertheless, it might be applicable for redox-controlled metal-sensing [60–62].

On the contrary, **NDIC8** forms pseudo[2]rotaxanes and facilitates the synthesis of a [2]rotaxane. Due to the remote position of the NDI unit and the rigid linker, rotaxane formation does not have a significant impact on the optoelectronic properties of the NDI moiety. The electrostatic interaction observed for NDI^2−^ and ammonium ions observed in the pseudo[2]rotaxane **A1·**PF_6_@**NDIC8**, which even results in dethreading of the pseudorotaxane, may give rise to new switching modes in more complex molecular structures.

Overall, these findings demonstrate the benefit of detailed and systematic studies on the noncovalent interactions cohering the components of switchable MIMs for the construction of new architectures with emergent properties.

## Supporting Information

File 1 Experimental section, including synthetic procedures, copies of NMR spectra, ITC, electrochemical, mass spectrometric and spectroelectrochemical data.

File 2 Crystallographic data (cif) for **exTTFC7**, **NDIC7**, and **NDIC8**.
