# Supplementary material for: Thermodynamic and electrochemical study of tailor-made crown ethers for redox-switchable (pseudo)rotaxanes
Source: Beilstein J Org Chem. 2020 Oct 20;16:2576–88. doi: 10.3762/bjoc.16.209 (PMC7590624; doi:10.3762/bjoc.16.209)
Supplement: File 1 — Experimental section, including synthetic procedures, copies of NMR spectra, ITC, electrochemical, mass spectrometric and spectroelectrochemical data. [file Beilstein_J_Org_Chem-16-2576-s001.pdf]

BEILSTEIN JOURNAL OF ORGANIC CHEMISTRY

Supporting Information

for

Thermodynamic and electrochemical study of tailor-made crown ethers for redox-switchable (pseudo)rotaxanes

Henrik Hupatz, Marius Gaedke, Hendrik V. Schröder, Julia Beerhues, Arto Valkonen, Fabian Klautzsch, Sebastian Müller, Felix Witte, Kari Rissanen, Biprajit Sarkar and Christoph A. Schalley

Beilstein J. Org. Chem. **2020,** *16,* 2576–2588. doi:10.3762/bjoc.16.209

Experimental section, including synthetic procedures, copies of NMR spectra, ITC, electrochemical, mass spectrometric and spectroelectrochemical data

License and Terms: This is a supporting information file under the terms of the Creative Commons Attribution License (https://creativecommons.org/licenses/by/4.0). Please note that the reuse, redistribution and reproduction in particular requires that the authors and source are credited.
The license is subject to the *Beilstein Journal of Organic Chemistry* terms and conditions: (https://www.beilstein-journals.org/bjoc)

Table of contents

1. Experimental details………………………………………………………………………. S1

1.1. General methods……………………………………………………………. S1

1.2. Synthetic Procedures............……………………….................................S1

2. Crystallographic Data……………………………………………………………………S10

3. Isothermal Titration Calorimetry………………………………………………………..S14

4. Electrochemical measurements………………………………………………………S17

5. Rotaxane characterization……………………………………………………………. S21

5.1. 2D NMR spectroscopy……………………………………………………. S21

5.2. Tandem mass spectrometry...………………………................................. S23

6. Spectroelectrochemical Measurements……………………………………………...S25

7. ^{1}H and ^{13}C NMR spectra……………………………………………………….............. S27

8. References………………………………………………………………………….........S39

1. Experimental details

1.1. General Methods

All reagents and solvents were obtained from commercial sources and used without further purification. Dry solvents were purchased from Acros Organics or obtained from the M. BRAUN Solvent purification system SPS 800. 2,3-bis(2-cyanoethylthio)-6,7-bis(methylthio)tetrathia-fulvalene [1], diiodide **1** [2], **BC7** [3], ditosylate **5** [4], monoanhydride **7** [5], (4-(prop-2-yn-1-yl-oxy)phenyl)methanaminium chloride [6], **A2**·PF_6 [7], **A1**·$BArF_{24}$ [8], and 2,6-dimethoxy-benzonitrile oxide stopper **St1** [9], were synthesized according to literature procedures. Thin-layer chromatography was performed on silica gel-coated plates with fluorescent indicator F254 (Merck). For column chromatography, silica gel (0.04–0.063 mm, Merck) was used.

^{1}H and ^{13}C NMR experiments were performed on JEOL ECX 400, JEOL ECP 500, Bruker AVANCE 500 or Bruker AVANCE 700 instruments. Residual solvent signals were used as the internal standards. All shifts are reported in ppm and NMR multiplicities are abbreviated as s (singlet), d (doublet), t (triplet), m (multiplet), and br (broad). Coupling constants *J* are reported in hertz. Compounds containing the tetrakis[3,5-bis(trifluoromethyl)phenyl]borate ($BArF_{24}^{-}$) anion show ^{13}C NMR spectra with ^{19}F, ^{10}B, and ^{11}B couplings. These signals were denoted as one signal.

Electrospray ionization high-resolution mass spectra (HRMS) were measured on an Agilent 6210 ESI-TOF device.

1.2. Synthetic procedures

TTFC7

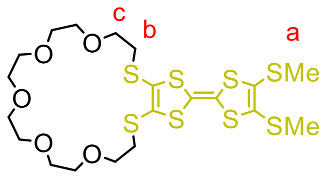

2,3-Bis(2-cyanoethylthio)-6,7-bis(methylthio)tetrathiafulvalene (50.0 mg, 0.11 mmol, 1 equiv) was dissolved in dry DMF (4 mL) and $CsOH{\cdot}H_2O$ (35.8 mg, 0.22 mmol, 2 equiv) dissolved in dry MeOH (1.5 mL) was added dropwise over 30 min under an Ar atmosphere. After stirring for 1 h, the resulting dark red solution was dropped to a solution of diiodide **1** (53.7 mg, 0.11 mmol, 1 equiv) in DMF (20 mL) in an ice bath over 1 h. The mixture was stirred in the thawing ice bath overnight. Afterwards, the solvent was removed under reduced pressure and the resulting residue was dissolved in CH_2Cl_2 (50 mL). The organic layer was washed with water (50 mL) and brine (50 mL). After drying over $MgSO_4$, the solution was concentrated under reduced pressure and the residue was purified by column chromatography (SiO_2, CH_2Cl_2

→ CH_2Cl_2/MeOH 100:1, R_f ≈ 0.6 in CH_2Cl_2/MeOH 50:1). The desired product (45 mg, 0.07 mmol, 69%) was obtained as dark red oil.

^{1}H NMR (700 MHz, CD_2Cl_2): δ = 2.42 (s, 6H, a), 3.03 (t, ^3J = 6.2 Hz, 4H, b), 3.62 – 3.61 (m, 16H, OCH_2), 3.69 (t, ^3J = 6.2 Hz, 4H, c) ppm. **^{13}C NMR** (176 MHz, CD_2Cl_2): δ = 19.6, 36.4, 70.2, 71.2, 71.2, 71.3, 71.3, 111.2, 128.1, 128.7 ppm. **ESI-HRMS** (CH_3CN): m/z calcd. for [$C_{20}H_{30}O_5S_8$]: $[M]^+$ 605.9853, found: 605.9843; calcd. for $[M+Na]^+$ 628.9751, found: 628.9734; calcd. for $[M+K]^+$ 644.9490, found: 644.9492.

21,22-Diiodo-2,3,5,6,8,9,11,12,14,15,17,18-dodecahydrobenzo[*b*][1,4,7,10,13,16,19]-heptaoxacyclohenicosine (**2**)

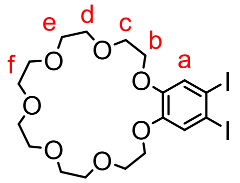

Periodic acid (231 mg, 1.01 mmol, 1.8 equiv) and iodine (114 mg, 0.45 mmol, 0.8 equiv) were dissolved in dry MeOH (5 mL) and stirred for 5 min under argon at rt. Afterwards, **BC7** (200 mg, 0.56 mmol, 1.0 equiv) dissolved in dry MeOH (7 mL) was added to the mixture and stirred for 12 h at 70 °C. The reaction mixture was quenched with a saturated aqueous $Na_2S_2O_3$ solution. The solvent was removed under reduced pressure and the residue dissolved in CH_2Cl_2 (50 mL). The organic phase was washed three times with water and brine. The organic phase was dried over $MgSO_4$ and the solvent was removed to isolate the desired product **2** (240 mg, 0.4 mmol, 66%) as light-yellow oil.

^{1}H NMR (500 MHz, $CDCl_3$): δ = 3.64 – 3.68 (m, 8H, f), 3.70 – 3.73 (m, 4H, e), 3.75 – 3.78 (m, 4H, d), 3.87 – 3.90 (m, 4H, c), 4.09 – 4.12 (m, 4H, b), 7.28 (s, 2H, a) ppm. **^{13}C NMR** (176 MHz, $CDCl_3$) δ = 69.6, 69.6, 70.7, 71.1, 71.1, 71.3, 96.7, 124.4, 149.6. ppm. **ESI-HRMS** (CH_3CN): m/z calcd. for [$C_{18}H_{26}I_2O_7$]: 626.0106 $[M+NH_4]^+$ found: 626.0094; calcd. for $[M+Na]^+$ 630.9660, found: 646.9644; calcd. for $[M+K]^+$ 646.9399, found: 646.9397.

7,8,10,11,13,14,16,17,19,20,22,23-Dodecahydro[1,3]dithiolo[4'',5'':5',6'][1,4]dithiino-[2',3':4,5]benzo[1,2-*b*][1,4,7,10,13,16,19]heptaoxacyclohenicosine-2-thione (**3**)

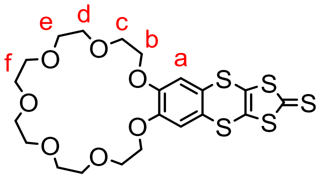

The diiodide **2** (235 mg, 0.38 mmol, 1.0 equiv), $(NEt_4)_2[Zn(dmit)_2]$ (dmit = 1,3 dithiole-2-thione-4,5-dithiolate (183 mg, 0.19 mmol, 0.5 equiv), Cu_2O (7 mg, 0.05 mmol, 0.1 equiv), and ethyl

acetoacetate (50 µL, 0.4 mmol, 1.0 equiv) were suspended in dry DMF (10 mL) and stirred for 12 h at 80 °C. Afterwards, the solvent was removed under reduced pressure and the residue was dissolved in CH_2Cl_2 (50 mL). The organic phase was washed three times with water and brine. The organic phase was dried over $MgSO_4$ and the solvent was removed. The crude product was purified via column chromatography (SiO_2, $CH_2Cl_2 \rightarrow CH_2Cl_2$/EtOH 20:1, $R_f \approx 0.3$ in CH_2Cl_2/EtOH 20:1) to isolate the desired thione **3** as yellowish powder in 58% yield (121 mg, 0.22 mmol).

^{1}H NMR (500 MHz, $CDCl_3$): δ = 3.64 – 3.68 (m, 8H, f), 3.71 – 3.73 (m, 4H, e), 3.76 – 3.79 (m, 4H, d), 3.90 – 3.93 (m, 4H, c), 4.13 – 4.17 (m, 4H, b), 6.97 (s, 2H, a) ppm. **^{13}C NMR** (176 MHz, $CDCl_3$) *δ* = 69.7, 69.9, 70.7, 71.2, 71.2, 71.4, 114.6, 125.8, 131.9, 149.7, 212.7 ppm. **ESI-HRMS** (MeOH): m/z calcd. for [$C_{21}H_{26}O_7S_5$]: $[M+NH_4]^+$ 568.0620 found: 568.0631; calcd. for $[M+Na]^+$ 573.0174, found: 573.0185; calcd. for $[M+K]^+$ 588.9914, found: 588.9926.

7,8,10,11,13,14,16,17,19,20,22,23-Dodecahydro[1,3]dithiolo[4'',5'':5',6'][1,4]dithiino[2',3':4,5]-benzo[1,2-*b*][1,4,7,10,13,16,19]heptaoxacyclohenicosine-2-one (**4**)

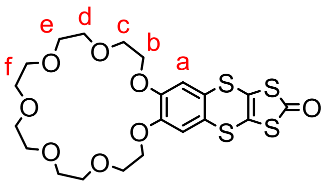

The thione **3** (58 mg, 0.11 mmol, 1 equiv) and mercury(II) acetate (101 mg, 0.33 mmol, 3 equiv) were dispersed in a mixture of $CHCl_3$ (5 mL) and acetic acid (2 mL). After stirring for 2 days at rt the reaction mixture was filtered through a celite pad, diluted with $CHCl_3$, and quenched with saturated aq. $NaHCO_3$ (20 mL). The organic phase was washed with brine, dried over $MgSO_4$, and the solvent was removed. The desired product **10** could be isolated as yellow powder in 95% yield (53 mg, 0.10 mmol).

^{1}H NMR (600 MHz, $CDCl_3$): δ = 3.63 – 3.67 (m, 8H, f), 3.69 – 3.72 (m, 4H, e), 3.75 – 3.78 (m, 4H, d), 3.89 – 3.92 (m, 4H, c), 4.13 – 4.17 (m, 4H, b), 6.97 (s, 2H, a) ppm. **^{13}C NMR** (126 MHz, $CDCl_3$) *δ* = 69.6, 69.8, 70.7, 71.1, 71.2, 71.3, 114.3, 122.0, 126.1, 149.7, 191.4 ppm. **ESI-HRMS** (MeOH): m/z calcd. for [$C_{21}H_{26}O_8S_4$]: $[M+K]^+$ 573.0142, found: 573.0126.

exTTFC7

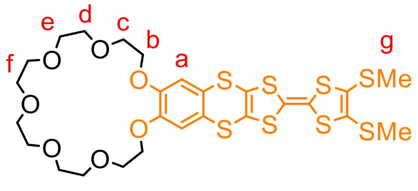

The one **4** (53 mg, 0.10 mmol, 1.0 equiv) and 4,5-bis(methylthio)-1,3-dithiole-2-thione (25 mg, 0.11 mmol, 1.1 equiv) were dissolved in $P(OEt)_3$ (20 mL). After stirring for 2 h at 80°C the solvent was removed in oil pump vacuum and the residue was dissolved in CH_2Cl_2 (50 mL). The crude product was purified by preparative thin layer chromatography plate (2000 µm, SiO_2, CH_2Cl_2/EtOH 30:1, $R_f \approx 0.4$ in CH_2Cl_2/EtOH 20:1) to isolate the desired product **exTTFC7** as yellow powder in 85% yield (61 mg, 0.09 mmol).

^{1}H NMR (700 MHz, CD_2Cl_2): δ = 2.42 (s, 6H, g), 3.59 – 3.61 (m, 8H, f), 3.63 – 3.65 (m, 4H, e), 3.68 – 3.71 (m, 4H, d), 3.83 – 3.86 (m, 4H, c), 4.10 – 4.12 (m, 4H, b), 6.94 (s, 2H, a) ppm. **^{13}C NMR** (176 MHz, $CDCl_3$) δ = 19.6, 70.0, 70.0, 71.1, 71.3, 71.4, 71.6, 113.3, 114.8, 116.7, 123.9, 127.0, 128.1, 149.7 ppm. **ESI-HRMS** (MeOH): m/z calcd. for [$C_{26}H_{32}O_7S_8$]: $[M+K]^+$ 750.9545, found: 750.9572.

2-(5,6,8,9,11,12,14,15,17,18-Decahydro-2H-naphtho[2,3-*h*][1,4,7,10,13,16,19]-hexaoxaazacyclohenicosin-10(3*H*)-yl)ethanamine (**6**)

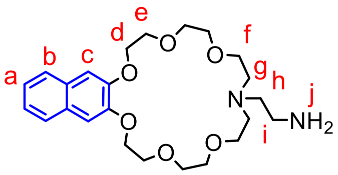

Ditosylate **5** (800 mg, 1.09 mmol, 1.0 equiv), EDABoc (174 mg, 1.09 mmol, 1.0 equiv), potassium iodide (262 mg, 1.58 mmol, 1.5 equiv), and K_2CO_3 (1506 mg, 10.9 mmol, 10 equiv) were dissolved in acetonitrile (25 mL) and heated to reflux overnight. After cooling to rt and filtration, the solvent was removed under reduced pressure and the residue was purified by column chromatography (SiO_2, CH_2Cl_2→ CH_2Cl_2/MeOH 10:1, $R_f \approx 0.1$ in CH_2Cl_2) to obtain pure protected amine (410 mg, 0.75 mmol, 69%) as pale yellow oil.

The protected amine was dissolved in CH_2Cl_2/trifluoroacetic acid (3:2, 2.5 mL) and stirred for 2 h at rt. The solution was concentrated under reduced pressure and the residue was solved in saturated $NaHCO_3$ solution (40 mL). The aqueous phase was extracted with CH_2Cl_2 (3 × 40 mL) and the combined organic phases were dried over $MgSO_4$. After removing the solvent, the crude product was purified by column chromatography (SiO_2, CH_2Cl_2 → CH_2Cl_2/MeOH 20:1, $R_f \approx 0.3$ in CH_2Cl_2/MeOH 20:1) to yield the desired product (204 mg, 0.46 mmol, 62%) as colorless oil. The product was obtained in an overall yield of 43%.

^{1}H NMR (500 MHz, $CDCl_3$): δ = 2.16 (s, 2H, j), 2.75 – 2.77 (m, 4H, g), 2.78 (br, 4H, h,i), 3.54 – 3.56 (m, 4H, f), 3.71 – 3.72 (m, 4H, OCH_2), 3.78 – 3.79 (m, 4H, OCH_2), 4.00 – 4.02 (m, 4H, e), 4.30 – 4.32 (m, 4H, d), 7.27 (s, 2H, c), 7.35 (AA'XX' spin system, $J_{AA'XX'}$ = 3.3, 6.2 Hz, 2H, a), 7.67 (AA'XX' spin system, $J_{AA'XX'}$ = 3.3, 6.2 Hz, 2H, b) ppm. **^{13}C NMR** (176 MHz, $CDCl_3$) δ

= 38.9, 51.8, 56.2, 68.6, 69.6, 69.9, 70.5, 70.6, 108.6, 124.9, 126.6, 129.5, 148.0 ppm. **ESI-HRMS** (CH_3CN): m/z calcd. for [$C_{24}H_{36}O_6N_2$]: $[M+H]^+$ 449.2646, found: 449.2661.

NDIC7

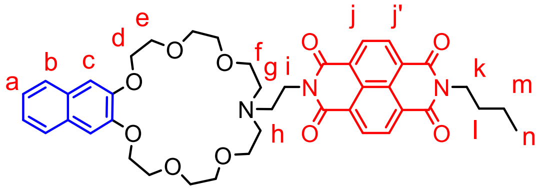

To a solution of amine **6** (170 mg, 0.38 mmol, 1.1 equiv) in DMF (2 mL) was added dropwise a solution of monoanhydride **7** (107 mg, 0.35 mmol, 1.0 equiv) in DMF (1 mL). The solution was stirred at 120 °C under Ar in a sealed tube overnight. After cooling to rt, the solvent was removed and the residue was purified by column chromatography (SiO_2, CH_2Cl_2/MeOH 20:1 → 10:1, $R_f \approx 0.4$ in CH_2Cl_2/MeOH 10:1) to yield the desired product **NDIC7** (158 mg, 0.21 mmol, 60%) as orange solid.

^{1}H NMR (500 MHz, $CDCl_3$): δ = 1.00 (t, ^3J = 7.4 Hz, 3H, n), 1.43 – 1.51 (m, 2H, m), 1.70 – 1.76 (m, 4H, l), 2.80 – 2.83 (m, 2H, h), 2.87 – 2.89 (m, 4H, g), 3.66 (br, 4H, f) 3.71 – 3.73 (m, 4H, OCH_2), 3.78 – 3.80 (m, 4H, OCH_2), 3.96 – 3.97 (m, 4H, e), 4.14 – 4.17 (m, 6H, d,k), 4.28 – 4.31 (m, 2H, i), 6.76 (s, 2H, c), 7.00 – 7.02 (m, 2H a), 7.24 – 7.26 (m, 2H, b), 8.56 – 8.60 (m, 4H, j, j') ppm. **^{13}C NMR** (176 MHz, $CDCl_3$) *δ* = 14.0, 20.5, 30.3, 39.4, 40.8, 52.3, 55.7, 68.8, 69.8, 70.2, 71.0, 71.2, 107.7, 123.8, 126.1, 126.5, 126.5, 126.5, 126.6, 128.9, 130.5, 130.7, 148.9, 162.8, 162.9 ppm. **ESI-HRMS** (CH_3CN): m/z calcd. for [$C_{42}H_{47}N_3O_{10}$]: $[M+H]^+$ 754.3334 found: 754.3361; calcd. for $[M+Na]^+$ 776.3154, found: 776.3180; calcd. for $[M+K]^+$ 792.2893, found: 792.2922.

tert-Butyl (3,5-dihydroxyphenyl)carbamate (**8**)

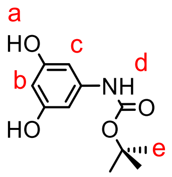

Aqueous NH_3 (28%, 100 mL) was purged with argon for 20 min. Benzene-1,3,5-triol (20.0 g, 0.16 mol, 2 equiv) was added and the mixture was stirred at rt for 2 h. The solvent was removed at reduced pressure, to the residue, toluene was added and removed at reduced pressure. The crude product was suspended in THF (150 mL) and Boc anhydride (17.5 g, 0.08 mol, 1 equiv) was added. The mixture was stirred under Ar overnight at rt and the solvent was

removed at reduced pressure. The crude product was purified by column chromatography (SiO_2, CH_2Cl_2/MeOH 15:1) and yielded a white solid (8.8 g, 0.04 mol, 25%).

^{1}**H NMR** (700 MHz, DMSO-d$_6$): δ = 1.43 (s, 9H, e), 5.80 (s, 1H, b), 6.40 (s, 2H, c), 9.01 (br, 1H, d), 9.02 (br, 2H, a) ppm. ^{13}**C NMR** (176 MHz, DMSO-d$_6$) δ = 28.2, 78.7, 96.8, 96.6 140.9, 152.6, 158.3 ppm. **ESI-HRMS** (MeOH): m/z calcd. for [$C_{11}H_{15}NO_4$]: $[M+Na]^+$ 248.0893, found: 248.0884.

2,5,8,11,13,16,19,22-Octaoxa-1(2,3)-naphthalena-12(1,3)-benzenacyclodocosaphan-12^5-amine (**9**)

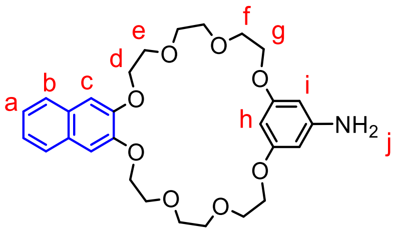

Cs_2CO_3 (5.0 g, 15.5 mmol, 5 equiv) was dissolved in acetonitrile (500 mL) and heated to reflux. A solution of ditosylate **5** (2.3 g, 3.1 mmol, 1 equiv) in acetonitrile (250 mL) and a solution of diol **8** (0.7 g, 3.1 mmol, 1 equiv) in acetonitrile (250 mL) were added dropwise and simultaneously. The mixture was stirred under reflux for 3 d. After cooling to rt and filtration, the solvent was removed under reduced pressure. The residue was dissolved in CH_2Cl_2 (300 mL) and washed three times with water and brine. After evaporation of the solvent, the crude product was purified by column chromatography (SiO_2, pentane/EtOAc/Et_3N 300:200:1 → 50:450:1, R_f ≈ 0.7 in 50:450:1) to obtain pure protected amine (1.1 g, 1.7 mmol, 55%) as yellow solid.

The protected amine and trifluoroacetic acid (2.6 mL, 34 mmol, 20 equiv) were dissolved in CH_2Cl_2 (100 mL) at 0 °C. The solution was warmed to rt and stirred for 16 h. The solvent was removed under reduced pressure and the residue was dissolved in saturated $NaHCO_3$ solution (200 mL). The aqueous phase was extracted with CH_2Cl_2 (3 × 200 mL). The combined organic phases were washed with water and dried over $MgSO_4$. After removing of the solvent, the crude product was purified by column chromatography (SiO_2, EtOAc/Et_3N 10:1, R_f ≈ 0.3) to yield the desired product (0.86 g, 1.7 mmol, quantitative) as pale yellow solid. The product was obtained in an overall yield of 55%.

^{1}**H NMR** (700 MHz, $CDCl_3$): δ = 3.61 (s, 2H, j), 3.72 – 3.73 (m, 4H, OCH_2), 3.77 – 3.78 (m, 4H, OCH_2), 3.79 – 3.80 (m, 4H, f), 3.93 – 3.95 (m, 4H, e), 4.07 – 4.09 (m, 4H, g), 4.24 – 4.26 (m, 4H, d), 5.85 (d, 2H, ^4J = 2.2 Hz, i), 6.12 (t, 1H, ^4J = 2.2 Hz, h), 7.15 (s, 2H, c), 7.31 (AA'XX' spin system, $J_{AA'XX'}$ = 3.3, 6.1 Hz, 2H, a), 7.65 (AA'XX' spin system, $J_{AA'XX'}$ = 3.3, 6.1 Hz, 2H, b) ppm. ^{13}**C NMR** (176 MHz, $CDCl_3$) δ = 68.0, 68.9, 69.8, 70.0, 71.1, 71.3, 93.3, 95.5, 109.0, 123.3, 126.4, 129.5, 148.4, 149.2, 160.9 ppm. **ESI-HRMS** (CH_3CN): m/z calcd. for

$[C_{24}H_{36}O_8N_1]$: $[M+Na]^+$ 536.2255, found: 536.2276; calcd. for $[M+K]^+$ 552.1994, found: 552.2011.

NDIC8

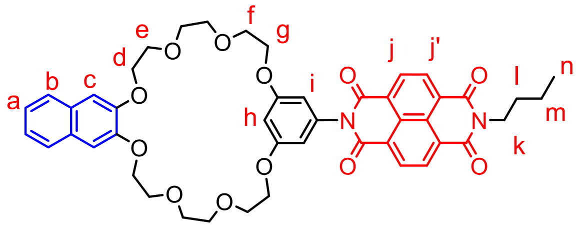

Monoanhydrate **7** (510 mg, 1.6 mmol, 1.1 equiv) was dissolved in dry DMF (55 mL) and stirred at 70 °C for 10 minutes in an Ar atmosphere. Et_3N (0.6 mL, 4.3 mmol, 3.0 equiv) and amine **9** (737 mg, 1.4 mmol, 1.0 equiv) were added and the solution was stirred at 70 °C for 24 h. The mixture was diluted in H_2O (200 mL) and extracted with EtOAc (2 × 200 mL). The solution was washed with brine and dried over MgSO4. The solvents were removed under reduced pressure and the crude product was purified by column chromatography (SiO_2, CH_2Cl_2/EtOAc 1:3, $R_f \approx$ 0.6 in EtOAc/Et_3N 40:1) to yield an orange solid (502 mg, 0.60 mmol, 43%)

^{1}H NMR (700 MHz, $CDCl_3$): δ = 1.00 (t, ^3J = 7.4 Hz, 3H, n), 1.47 (h, 2H, ^3J = 7.4 Hz, m), 1.72 – 1.77 (m, 2H, l), 3.74 – 3.75 (m, 4H, OCH_2), 3.80 – 3.81 (m, 4H, OCH_2), 3.83 – 3.84 (m, 4H, f) 3.95 – 3.97 (m, 4H, e), 4.16 – 4.17 (m, 4H, g), 4.20 – 4.21 (m, 2H, k), 4.26 – 4.28 (m, 4H, d), 6.47 (d, 2H, ^4J = 2.2 Hz, i), 6.89 (t, 1H, ^4J = 2.2 Hz, h), 7.14 (s, 2H, c), 7.29 (AA'XX' spin system, $J_{AA'XX'}$ = 3.3, 6.1 Hz, 2H, a), 7.62 (AA'XX' spin system, $J_{AA'XX'}$ = 3.3, 6.1 Hz, 2H, b), 8.74 (m, 4H, j, j') ppm. **^{13}C NMR** (176 MHz, $CDCl_3$) δ = 13.9, 20.5, 30.3, 40.9, 68.5, 68.8, 69.9, 70.1, 71.2, 71.3, 103.5, 108.6, 109.0, 124.3, 126.4, 126.8, 126.9, 127.0, 127.1, 129.5, 131.1, 131.4, 136.0, 149.3, 160.8, 162.8, 162.9 ppm. **ESI-HRMS** (CH_3CN): m/z calcd. for $[C_{46}H_{46}O_{12}N_2]$: $[M+NH_4]^+$ 836.3389 found: 836.3397; calcd. for $[M+Na]^+$ 841.2943, found: 841.2962; calcd. for $[M+K]^+$ 857.2682, found: 857.2701.

A1·PF_6

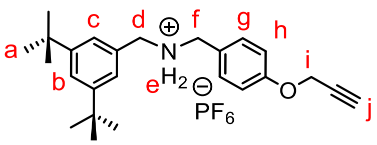

(4-(Prop-2-yn-1yloxy)phenyl)methanaminium chloride (473 mg, 2.4 mmol, 1 equiv) was dispersed in dry EtOH (40 mL) and Et_3N (242 mg, 2.4 mmol, 1 equiv) was added. Afterwards, 3,5-di-*tert*-butylbenzaldehyde (522 mg, 2.4 mmol, 1 equiv) was added and the mixture was stirred at reflux under Ar atmosphere for 3 h. The solution was cooled to 0 °C and $NaBH_4$ (454 mg, 12 mmol, 5 equiv) was added. After stirring at rt overnight, the suspension was

quenched with sat. $NaHCO_3$ solution. The mixture was filtered and the solid residue was washed with CH_2Cl_2 (150 mL). The combined filtrate was phase separated and the organic phase was washed with water (3 × 100 mL). It was dried with $MgSO_4$ and the solvent was removed at reduced pressure. The crude product was purified by column chromatography (SiO_2, CH_2Cl_2/MeOH 80:1 → 50:1) to yield a colorless oil.

The amine (745 mg, 2.0 mmol, 83 %) was dissolved in acetonitrile (10 mL), NH_4PF_6 (379 mg, 2.3 mmol) was added and argon was bubbled through the solution for 4 h. The solvent was removed at reduced pressure and the residue was suspended in water (100 mL). The suspension was extracted with Et_2O (3 × 100 mL) and the combined organic phases were washed with water (100 mL). After drying with $MgSO_4$, the solvent was removed at reduced pressure to yield the desired product **A1**•PF_6 (1010 mg, 2.0 mmol, quantitative) as white solid.

^{1}H NMR (700 MHz, $CDCl_3$): δ = 1.32 (s, 18H, a), 2.50 (t, ^4J = 2.4 Hz, 1H, j), 4.09 (s, 2H, f), 4.13 (s, 2H, d), 4.63 (d, ^4J = 2.4 Hz, 2H, i), 6.10 (br, 2H, e), 6.97 – 6.98 (m, 2H, h), 7.18 (d, ^4J = 1.8 Hz, 2H, c), 7.27 – 7.28 (m, 2H, g), 7.47 (t, ^4J = 1.8 Hz, 1H, b) ppm. **^{13}C NMR** (176 MHz, $CDCl_3$) *δ* = 31.4, 35.1, 50.7, 52.0, 55.9, 76.1, 78.1, 115.9, 122.4, 124.0, 124.2, 128.7, 131.5, 152.7, 158.9. **ESI-HRMS** (CH_3CN): m/z calcd. for [$C_{25}H_{34}F_6NOP$]: [M-PF_6]$^+$ 364.2635, found: 364.2639.

NDIC8Rot

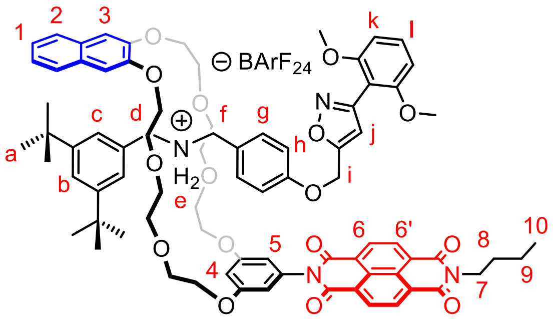

NDIC8 (28 mg, 34 µmol, 1.1 equiv) and **A1**•$BArF_{24}$ (38 mg, 31 µmol, 1.0 equiv) were dissolved in dry 1,2-dichloroethane (1.5 mL) and ultra-sonicated for 10 min. **St** (11 mg, 62 µmol, 2.0 equiv) was added and the solution was stirred at 50 °C for 3 days. The solvent was removed under reduced pressure and the crude product was purified by column chromatography (SiO_2, CH_2Cl_2/MeOH 100:1, R_f ≈ 0.4) and preparative thin layer chromatography plate (SiO_2, 500 µm, CH_2Cl_2/MeOH 100:1) to yield an orange solid (30 mg, 14 µmol, 45%).

^{1}H NMR (700 MHz, $CDCl_3$): δ = 1.00 (t, ^3J = 7.4 Hz, 3H, 10), 1.26 (s, 18H, a), 1.45 – 1.53 (m, 2H, 9), 1.73 – 1.77 (m, 2H, 8), 3.50 - 3.57 (m, 4H, OCH_2), 3.59 – 3.62 (m, 4H, OCH_2), 3.67 – 3.71 (m, 4H, OCH_2), 3.76 – 3.78 (m, 8H, OMe, OCH_2), 3.81 – 3.83 (m, 2H, OCH_2), 3.97 – 4.00 (m, 2H, OCH_2), 4.15 – 4.17 (m, 4H, OCH_2), 4.19 – 4.23 (m, 4H, 7, OCH_2), 4.38 – 4.40 (m, 4H,

i, f), 4.48 – 4.50 (m, 2H, d), 6.35 (s, 1H, j), 6.51 – 6.52 (m, 2H, h), 6.55 (d, ^{4}J = 2.2 Hz, 2H, 5), 6.64 (d, ^{3}J = 8.4 Hz, 2H, k), 6.67 (t, ^{4}J = 2.2 Hz, 1H, 4), 6.91 (s, 2H, 3), 7.14 – 7.15 (m, 2H, g), 7.35 – 7.39 (m, 5H, 1, c, l), 7.48 (t, ^{4}J = 1.8 Hz, 1H, b), 7.50 – 7.51 (m, 4H, $BArF_{24}$), 7.62 (AA'XX' spin system, $J_{AA'XX'}$ = 3.3, 6.1 Hz, 2H, 2), 8.56 – 8.60 (m, 4H, j), 7.69 – 7.70 (m, 8H, $BArF_{24}$), 7.73 (br, 2H, e), 8.75 (d, ^{3}J = 7.6 Hz, 2H, 6/6'), 8.78 (d, ^{3}J = 7.6 Hz, 2H, 6/6') ppm. **^{13}C NMR** (176 MHz, $CDCl_3$) δ = 13.9, 20.5, 30.3, 31.4, 35.1, 41.0, 52.4, 53.0, 56.2, 60.6, 68.0, 68.2, 69.4, 70.5, 70.9, 71.6, 104.3, 104.6, 106.6, 106.9, 108.0, 108.7, 114.8, 117.6, 123.2, 123.3, 124.1, 124.7, 125.7, 126.5, 127.0, 127.1, 127.3, 129.0, 130.5, 131.1, 131.1, 131.5, 131.5, 134.9, 137.1, 146.4, 152.3, 157.3, 158.7, 159.0, 160.5, 161.9, 162.8, 163.0, 165.7 ppm. **ESI-HRMS** (CH_3CN): m/z calcd. for [$C_{112}H_{101}BF_{24}N_4O_{16}$]: [M-$BArF_{24}$]$^+$ 1361.6274 found: 1361;6277.

.

2. Crystallographic data

General details. Single crystal X-ray data for **exTTFC7** were collected at 120 K on an Agilent Super-Nova dual wavelength diffractometer with a micro-focus X-ray source and multilayer optics monochromatized CuKα (λ = 1.54184 Å) radiation. Program *CrysAlisPro* [10] was used for collection and reduction of data. The diffraction intensities were corrected for absorption using analytical face index absorption correction method [11] for the data. The structures were solved with direct methods (*SHELXT*) [12] and refined by full-matrix least squares on F^2 using *SHELXL*-2018/3 [13]. Anisotropic displacement parameters were assigned to non-hydrogen atoms. All hydrogen atoms were constrained to their idealised positions and refined using riding models with U_{eq}(H) of 1.5U_{eq}(C) for terminal methyl groups, and of 1.2U_{eq}(C) for other groups. The summary details of crystal data, data collection, and the refinement results are documented below.

Single crystal X-ray data for **NDIC8** and **NDIC7** were collected on a Bruker D8 Venture system at 100(2) K using graphite-monochromated MoKα (λα = 0.71073 Å) and CuKα (λ = 1.54184 Å) radiation. The strategy for the data collection was evaluated using APEX3 software and the data were collected by the omega + phi scan techniques. The data were scaled and reduced using SAINT+ and SADABS software. The structure was solved by intrinsic phasing using SHELXT-2014/7. It was refined by full matrix least-squares using SHELXL-2014/7 and was refined on F^2. Non-hydrogen atoms were refined anisotropically [13-19].

Deposition numbers CCDC 1977518 (**exTTFC7**), 1902746 (**NDIC7**), and 1906086 (**NDIC8**) contains the supplementary crystallographic data for this paper. These data are provided free of charge by the joint Cambridge Crystallographic Data Centre and Fachinformationszentrum Karlsruhe Access Structures service www.ccdc.cam.ac.uk/structures.

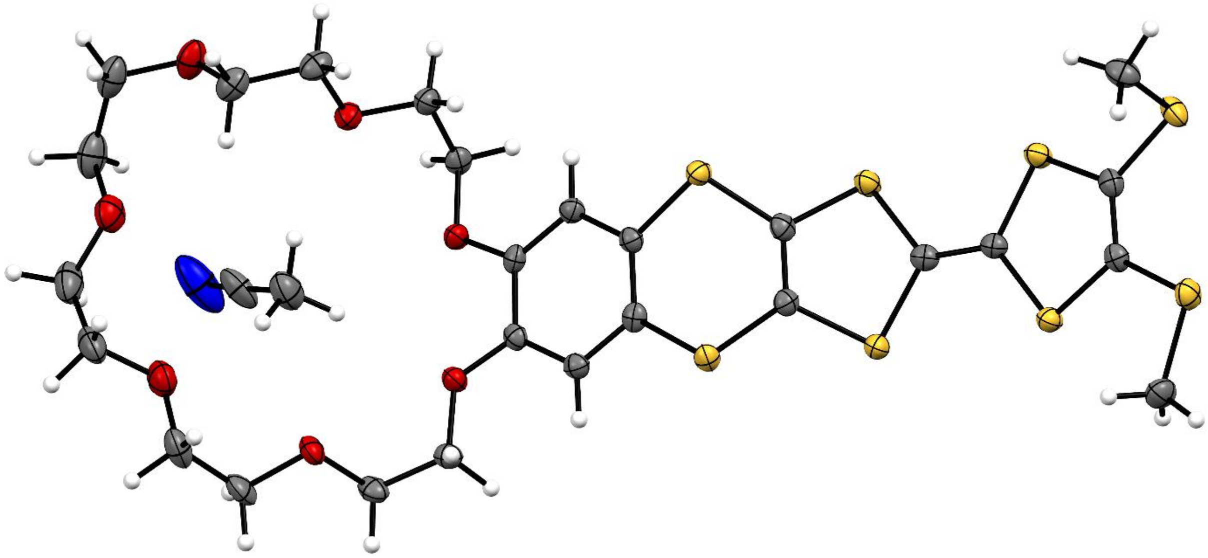

Figure S1: Thermal ellipsoid diagram of **exTTFC7** with 50% ellipsoid probability level. Colour codes: C (dark grey), H (white), N (blue), O (red) and S (yellow).

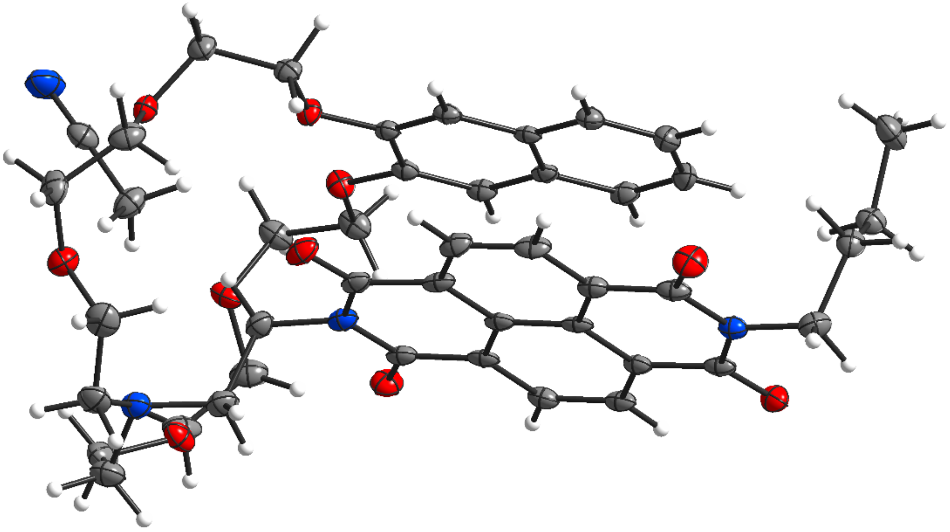

Figure S2: Thermal ellipsoid diagram of **NDIC7** with 50% ellipsoid probability level. Colour codes: C (dark grey), H (white), N (blue) and O (red).

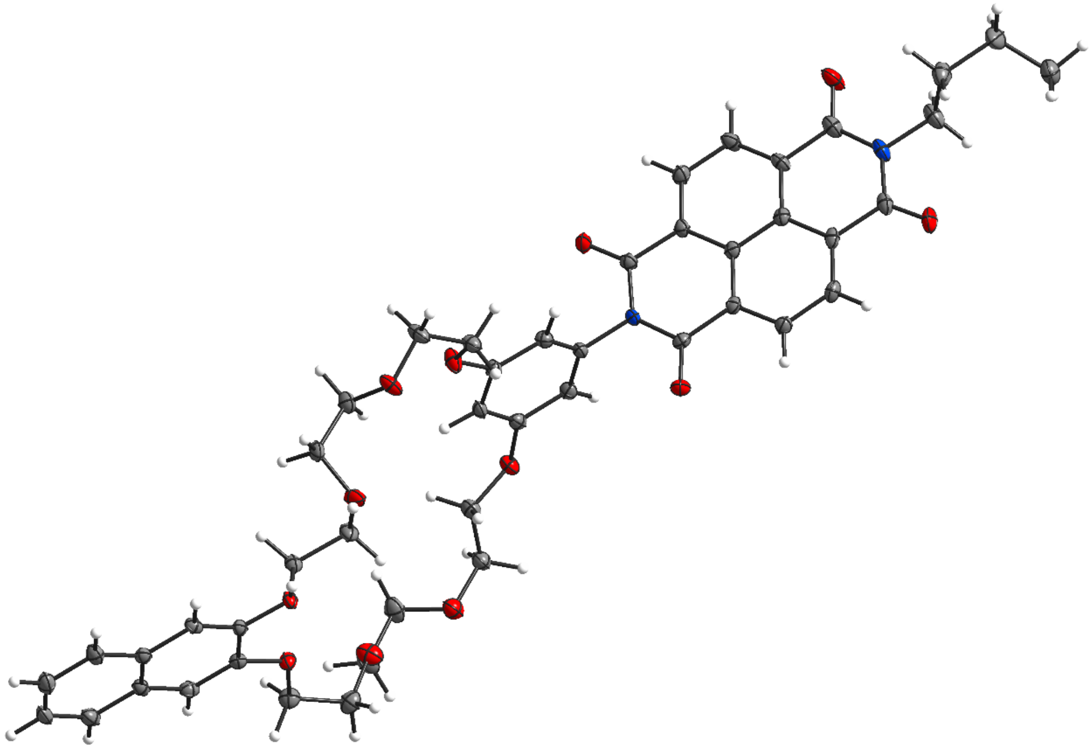

Figure S3: Thermal ellipsoid diagram of **NDIC8** with 50% ellipsoid probability level. Colour codes: C (dark grey), H (white), N (blue) and O (red).

Table S1: Crystallographic data.

compound	exTTFC7	NDIC7	NDIC8
chemical formula	$C_{28}H_{35}NO_7S_8$	$C_{44}H_{50}N_4O_{10}$	$C_{46}H_{46}N_2O_{12}$
*M*r / g mol^{-1}	754.05	794.88	818.85
crystal system	monoclinic	monoclinic	monoclinic
space group	*P2₁/c*	*P2₁/c*	*P2₁*
a / Å	31.5592(13)	13.6717(13)	19.695(4)
b / Å	12.8309(5)	9.8821(10)	9.1282(16)
c / Å	8.3107(3)	28.639(3)	21.618(4)
α / °	90	90	90
β / °	94.379(3)	90.657(4)	96.451(11)
γ / °	90	90	90
V / Å	3355.5(2)	3869.1(7)	3861.8(13)
Z	4	4	4
density / g cm^{-3}	1.493	1.365	1.408
F(000)	1576	1688	1728
radiation type	CuK_{α}	MoK_{α}	CuK_{α}
μ / mm^{-1}	5.317	0.097	0.845
crystal size / mm	0.12 x 0.09 x 0.05	0.42 x 0.22 x 0.10	0.31 x 0.09 x 0.07
meas refl.	13226	134093	125951
indep. refl.	6545	7104	16640
obsvd. [I > 2σ(I)] refl.	5274	5843	14307
max. and min. transmission	0.842, 0.629	0.745, 0.708	0.727, 0.654
R_{int}	0.0344	0.0678	0.0897
R [$F^2 > 2\sigma(F^2)$], wR(F^2), S	0.0487, 0.0860, 1.034	0.0522, 0.1254, 1.103	0.0449, 0.1064, 1.033
Δρmax, Δρmin (e Å^{-3})	0.487, -0.326	0.597, -0.285	0.663, -0.319

3. Isothermal titration calorimetry

ITC titrations were carried out in dry 1,2-dichloroethane (DCE) at 298 K on a TAM III microcalorimeter (Waters GmbH, TA Instruments, Eschborn, Germany). In a typical experiment, an 800 µL solution of the crown ether was placed in the sample cell at a concentration of 1.1 mM, and 250 µL of a solution of the ammonium salt (8.0 mM) were put into the syringe. The titrations consisted of 32 consecutive injections of 8 µL each with a 20 min interval between the injections. The heat of dilution was determined by titration of ammonium salt solutions into the sample cell containing blank solvent and were subtracted from each data set. The heat flow generated in the sample cell was measured as a differential signal between sample and reference cell. Hence, an exothermic event results in a positive and an endothermic in a negative heat flow. The data were analysed using the instrument's internal software package and fitted with a 1:1 binding model. Each titration was conducted at least three times and the measured values for K and ΔH were averaged.

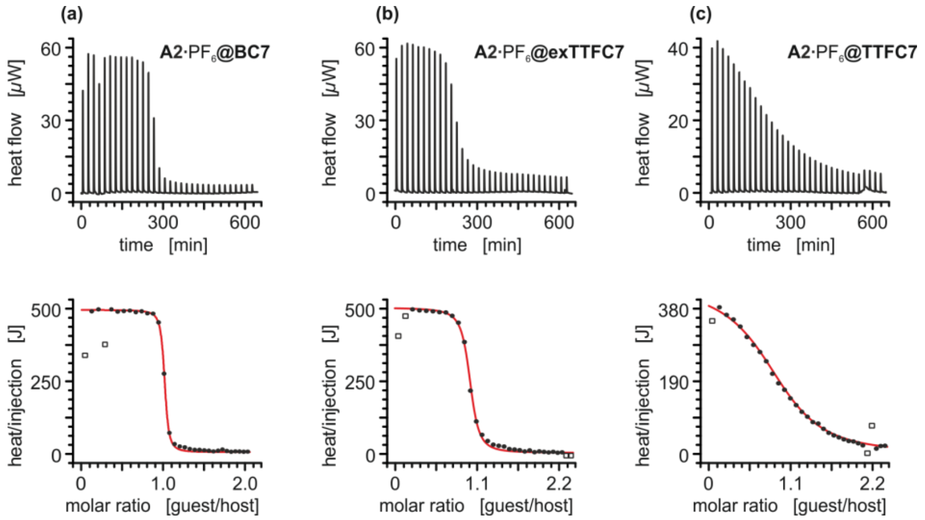

Figure S4: Titration plots (heat flow versus time and heat/volume versus guest/host ratio) obtained from ITC experiments at 298 K in 1,2-dichloroethane: (a) vial: **BC7**, syringe: axle **A2**•PF_6; (b) vial: **exTTFC7**, syringe: axle **A2**·PF_6; (c) vial: **TTFC7**, syringe: axle **A2**•PF_6. Points marked with non-filled squares were not considered in the fitting process.

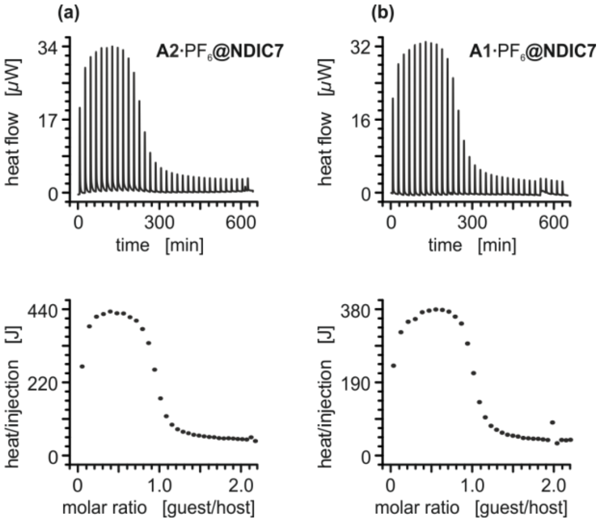

Figure S5: Titration plots (heat flow versus time and heat/volume versus guest/host ratio) obtained from ITC experiments at 298 K in 1,2-dichloroethane: (a) vial: **NDIC7**, syringe: axle **A2**•PF_6; (b) vial: **NDIC7**, syringe: axle **A1**•PF_6.The data points of the **A2**•PF_6@**NDIC7** do not agree with a 1:1 binding model. The increasing heat generated in the first 5 data points suggests a second process taking place in the mixture, which could be explained with the protonation of the tertiary amine of **NDIC7** by **A2**•PF_6. **A1**•PF_6 is sterically too big to thread **NDIC7** for a pseudo[2]rotaxane. Yet, the titration of **NDIC7** with the larger axle **A1**•PF_6 exhibits very similar results as with the smaller axle **A2**•PF_6. These results suggest that both ammonium axles undergo a similar type of reaction with **NDIC7**, which could be the protonation of the tertiary amine or the complexation in a non-threaded complex.

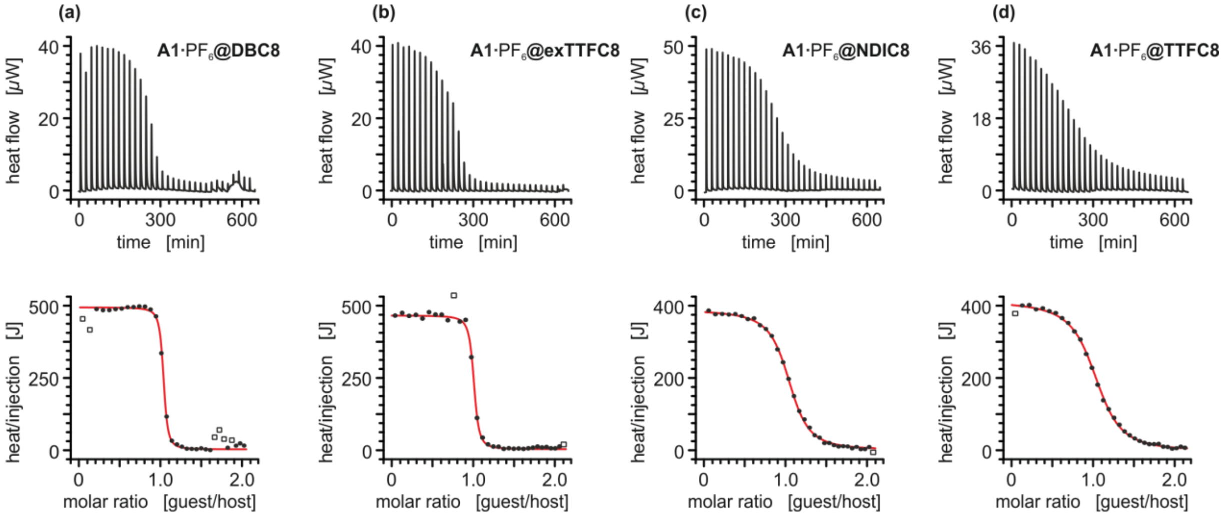

Figure S6: Titration plots (heat flow versus time and heat/volume versus guest/host ratio) obtained from ITC experiments at 298 K in 1,2-dichloroethane: (a) vial: **DBC8**, syringe: axle **A2**•PF_6; (b) vial: **exTTFC8**, syringe: axle **A2**•PF_6; (c) vial: **NDIC8**, syringe: axle **A2**•PF_6; (d) vial: **TTFC8**, syringe: axle **A2**•PF_6. Points marked with non-filled squares were not considered in the fitting process.

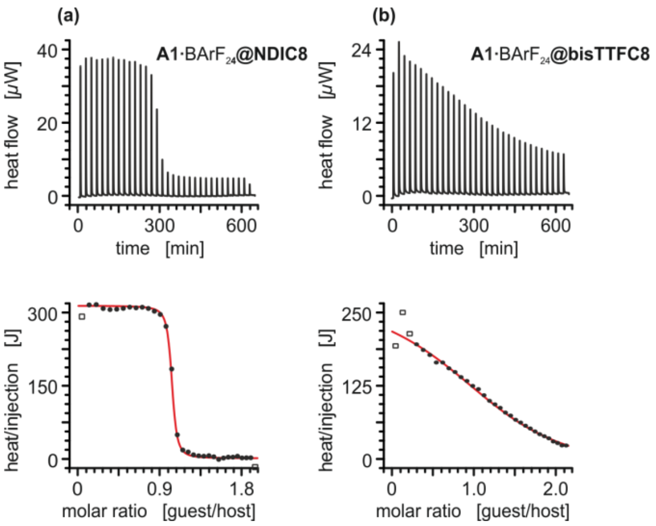

Figure S7: Titration plots (heat flow versus time and heat/volume versus guest/host ratio) obtained from ITC experiments at 298 K in 1,2-dichloroethane: (a) vial: **DBC8**, syringe: axle **A1**•PF_6; (b) vial: **exTTFC8**, syringe: axle **A1**•PF_6; (c) vial: **NDIC8**, syringe: axle **A1**•PF_6; (d) vial: **TTFC8**, syringe: axle **A1**•PF_6. Points marked with non-filled squares were not considered in the fitting process.

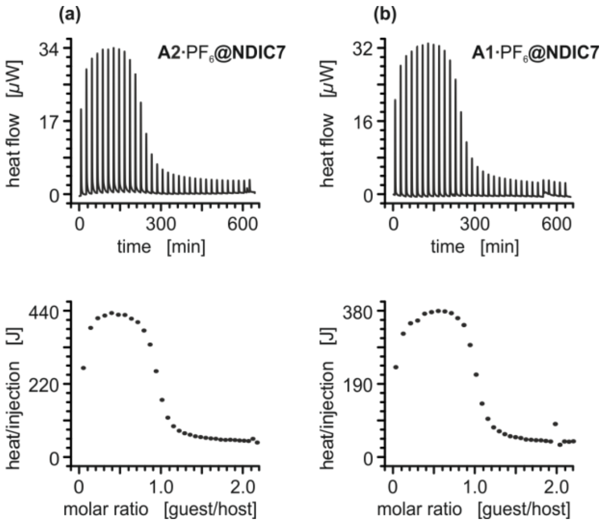

Figure S8: Titration plots (heat flow versus time and heat/volume versus guest/host ratio) obtained from ITC experiments at 298 K in 1,2-dichloroethane: (a) vial: **NDIC8**, syringe: axle **A1**•$BArF_{24}$; (b) vial: **bisTTFC8**, syringe: axle **A1**•$BArF_{24}$. Points marked with non-filled squares were not considered in the fitting process.

4. Electrochemical measurements

Cyclic voltammetry (CV) and differential pulse voltammetry (DPV) were performed on an Autolab PGSTAT302N potentiostat using a three-electrode configuration: a freshly polished glassy carbon working electrode, a platinum wire counterelectrode and a silver wire pseudoreference electrode. The decamethylferrocene/decamethylferrocenium ($FeCp^*{}_2^{(0/+)}$) couple was used as internal reference. Dry and argon purged solvents were used. Half-wave potentials were determined by DPV measurements and confirmed with CV (scan rate 100 mV s^{-1}), which also ensured reversibility of the reduction or oxidation processes. During experiments using axle **A1**•PF_6, an irreversible oxidation at ≈1.4 V vs. $FeCp^*{}_2^{(0/+)}$ was observed after addition of the axle. We address this signal to the oxidation of the axle phenol ether unit. The cyclic voltammogram traces of **NDIRot** were recorded with 10, 25, 50, 100, 250, 500, 1000, and 2500 mV/s scan rates, to ensure that the observed processes are reversible and diffusion limited. In order to obtain the correct half-wave potentials, $FeCp^{*0}$/$FeCp^{*+}$ was used as the reference. These values were later referenced to FeCp/$FeCp^+$ as described in the literature [20]. The raw data was treated with Nova 1.5 by Metrohm and the plots were made with Origin *8* by OriginLab.

Table S2: Electrochemical data obtained by differential pulse voltammetry.

entry	compound	solvent[a]	$E_{1/2}^{r2}$ / V[b]	$E_{1/2}^{r1}$ / V[b]	$E_{1/2}^{o1}$ / V[b]	$E_{1/2}^{o2}$ / V[b]
			reversible reductions		reversible oxidations	
1	**exTTFC7**	DCE/	/	/	0.67	0.95
2	**exTTFC7** + **A2**·PF_6[d]	CH_3CN	/	/	0.67	0.95
3	**exTTFC8**[c]	(1:1)	/	/	0.66	0.93
4	**exTTFC8** + **A1**·PF_6[d]		/	/	0.67	0.91
5	**TTFC7**		/	/	0.59	0.83
6	**TTFC7** + **A2**·PF_6[d]		/	/	0.60	0.84
7	**TTFC8**		/	/	0.59	0.83
8	**TTFC8** + **A1**·PF_6[d]		/	/	0.59	0.82
9	**bisTTFC8**[c]		/	/	0.57	0.93
10	**bisTTFC8** + **A1**·PF_6[d]		/	/	0.57	0.92
11	**NDIC7**		-0.96	-0.54	/	/
12	**NDIC7** + **A2**·PF_6[d]		-0.70	-0.46	/	/
13	**NDIC8**[c]		-0.95	-0.49	/	/
14	**NDIC8**[c] + **A1**·PF_6[d]		-0.72	-0.49	/	/
15	**NDIC8**[c] + $(CH_3)_2NH_2PF_6$[d]		-0.78	-0.45		
16	**NDIC8Rot**		-0.95	-0.50		
17	**exTTFC7**	DCE	/	/	0.65	1.01
18	**exTTFC7** + **A2**·PF_6[d]		/	/	0.67	1.01
19	**TTFC7**		/	/	0.59	0.87
20	**TTFC7** + **A2**·PF_6[d]		/	/	0.63	0.87
21	**bisTTFC8**		/	/	0.56	0.95
22	**NDIC8**[c]		-0.97	-0.53	/	/
23	**NDIC8Rot**		-0.96	-0.51	/	/

[a]With *n*-Bu_4NPF_6 (0.1 M) as the electrolyte. [b]Half-wave potentials are given against the decamethylferrocene/decamethylferrocenium couple as the reference; error = ± 0.01 V. [c]The compound showed only moderate solubility in the corresponding solvent. [d]Five equiv of the ammonium guest were added.

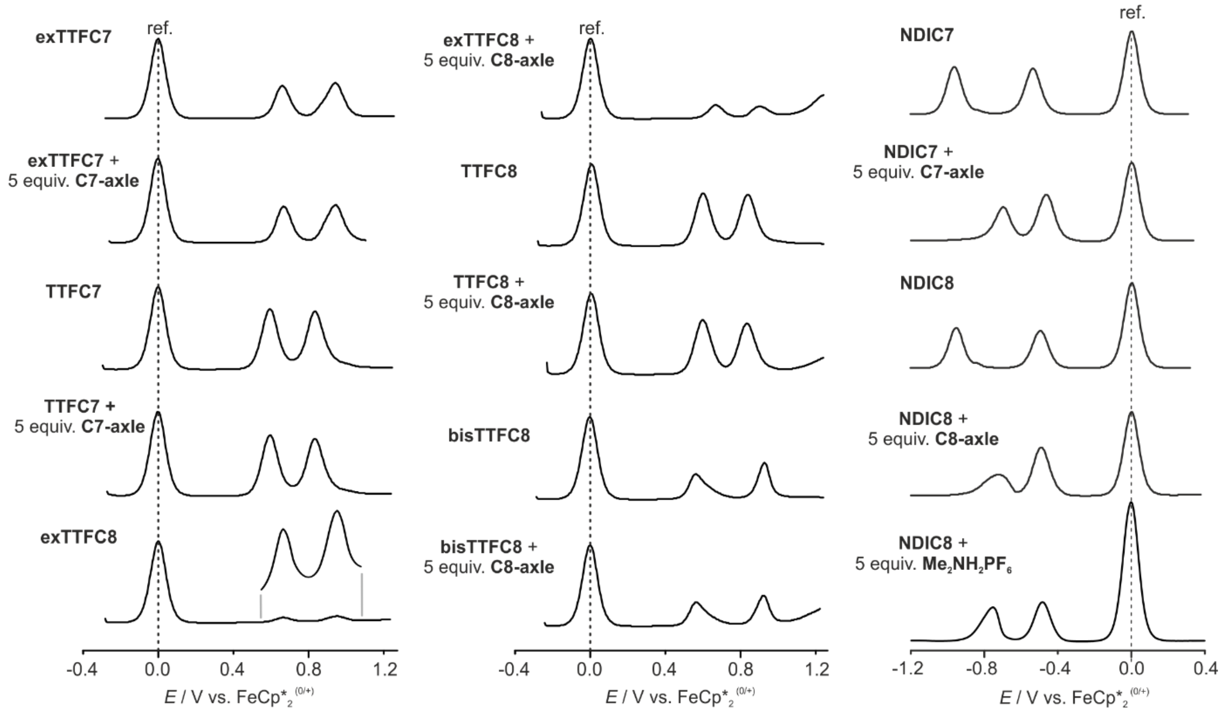

Figure S9: Differential pulse voltammograms (1 mM analyte, DCE/CH_3CN 1:1, 0.1 M *n*-Bu_4PF_6, 10 mV s^{-1} scan rate, 25 mV modulation amplitude, 50 ms modulation time, 5 mV step potential, 0.5 s interval time).

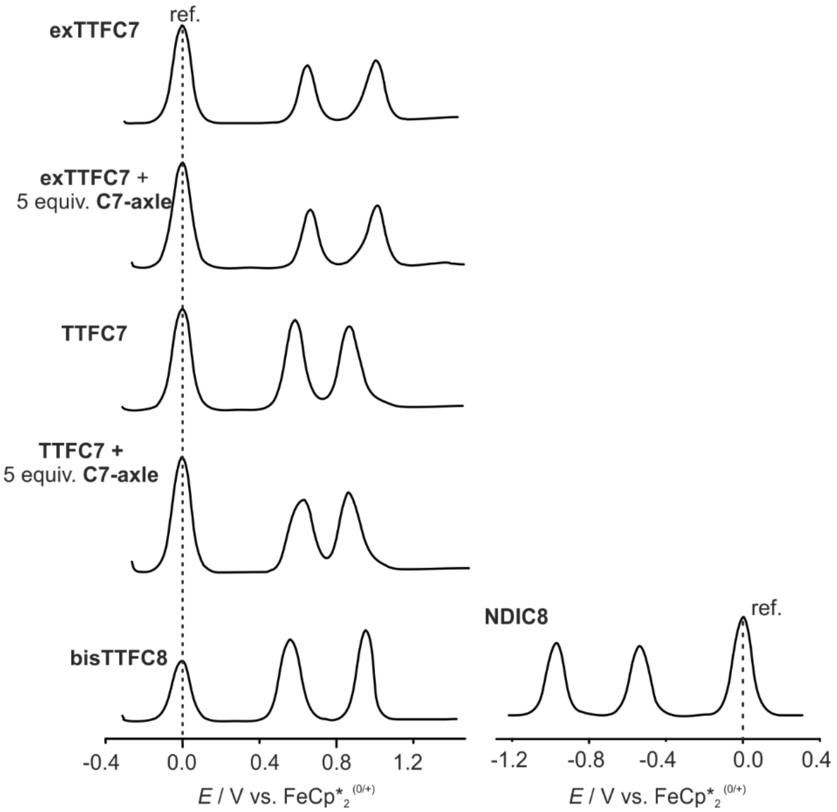

Figure S10: Differential pulse voltammograms (1 mM analyte, DCE, 0.1 M *n*-Bu_4PF_6, 10 mV s^{-1} scan rate, 25 mV modulation amplitude, 50 ms modulation time, 5 mV step potential, 0.5 s interval time).

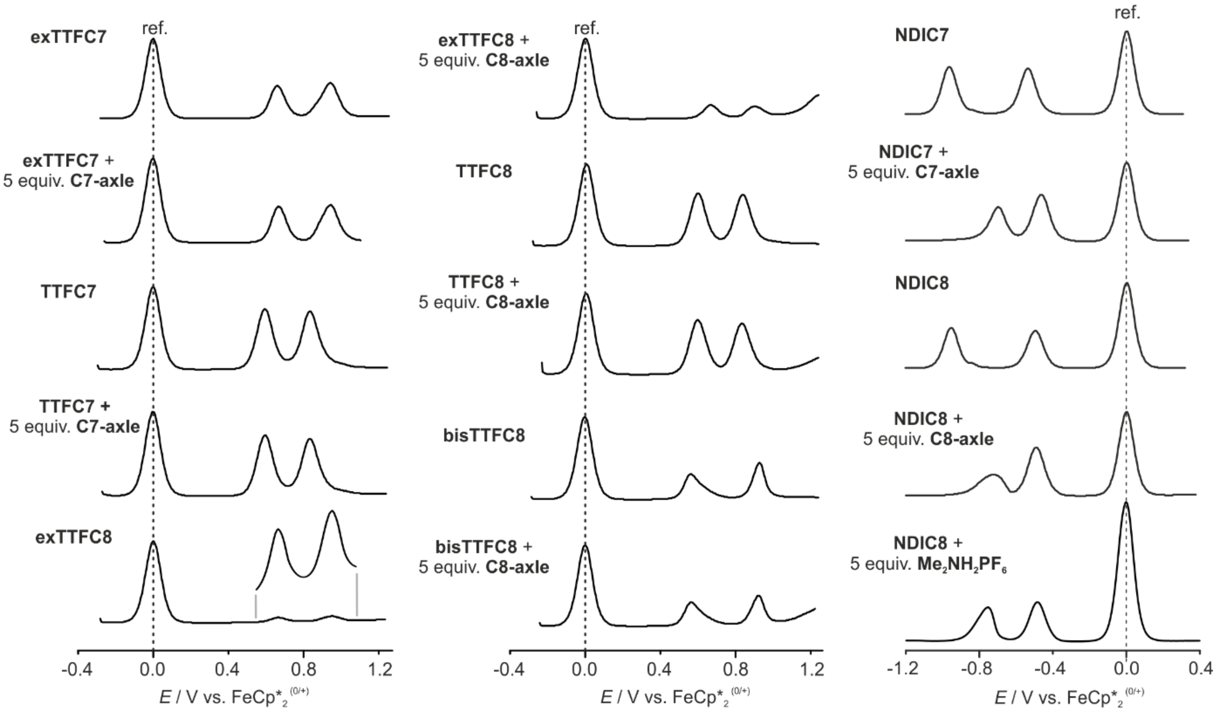

Figure S11: Differential pulse voltammogram (10 mV s^{-1} scan rate, 25 mV modulation amplitude, 50 ms modulation time, 5 mV step potential, 0.5 s interval time) and cyclic voltammogram (100 mV s^{-1}) of [2]rotaxane **NDIRot** (1.0 mM) in mixture of DCE/CH_3CN 1:1 and pure DCE (0.1 M *n*-Bu_4PF_6). The small signal at approximately −0.8 V was observed before adding the rotaxane and is addressed to an impurity. The decamethylferrocene/decamethylferrocenium couple was used as reference.

5. Rotaxane characterization

5.1. 2D NMR spectroscopy

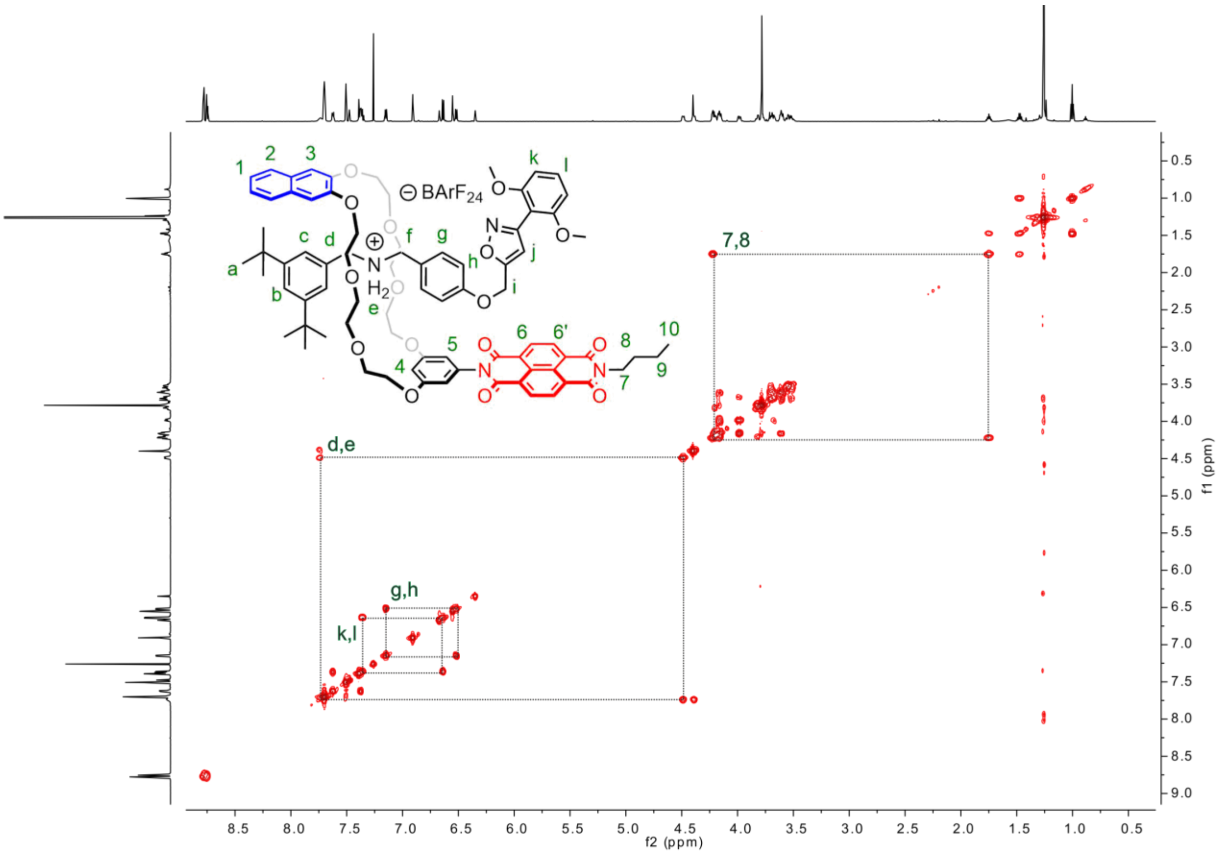

Figure S12: ^{1}H, ^{1}H COSY NMR spectrum (700 MHz, $CDCl_3$, 298 K) of rotaxane **NDIRot** with selected cross peaks for peak assignment.

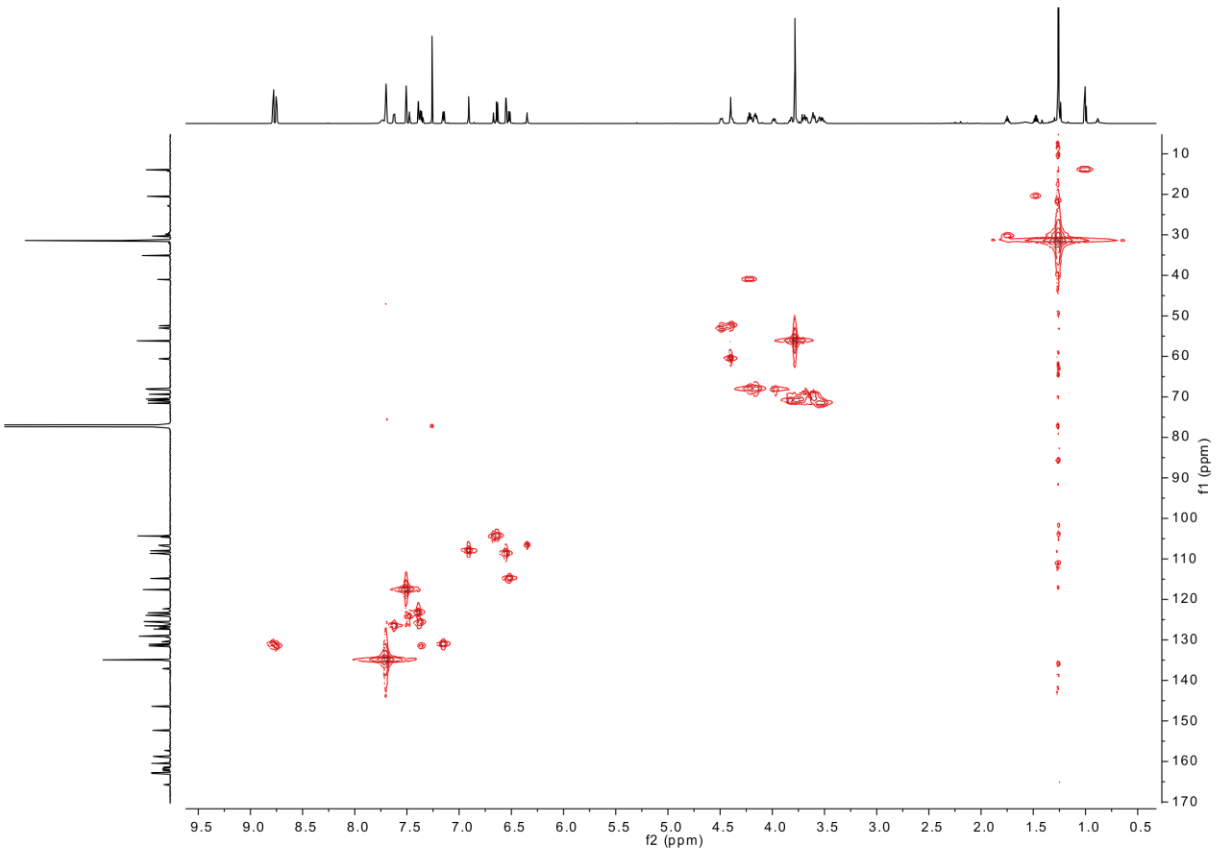

Figure S13: ^{1}H, ^{13}C HMQC NMR spectrum (700 MHz, $CDCl_3$, 298 K) of rotaxane **NDIRot**.

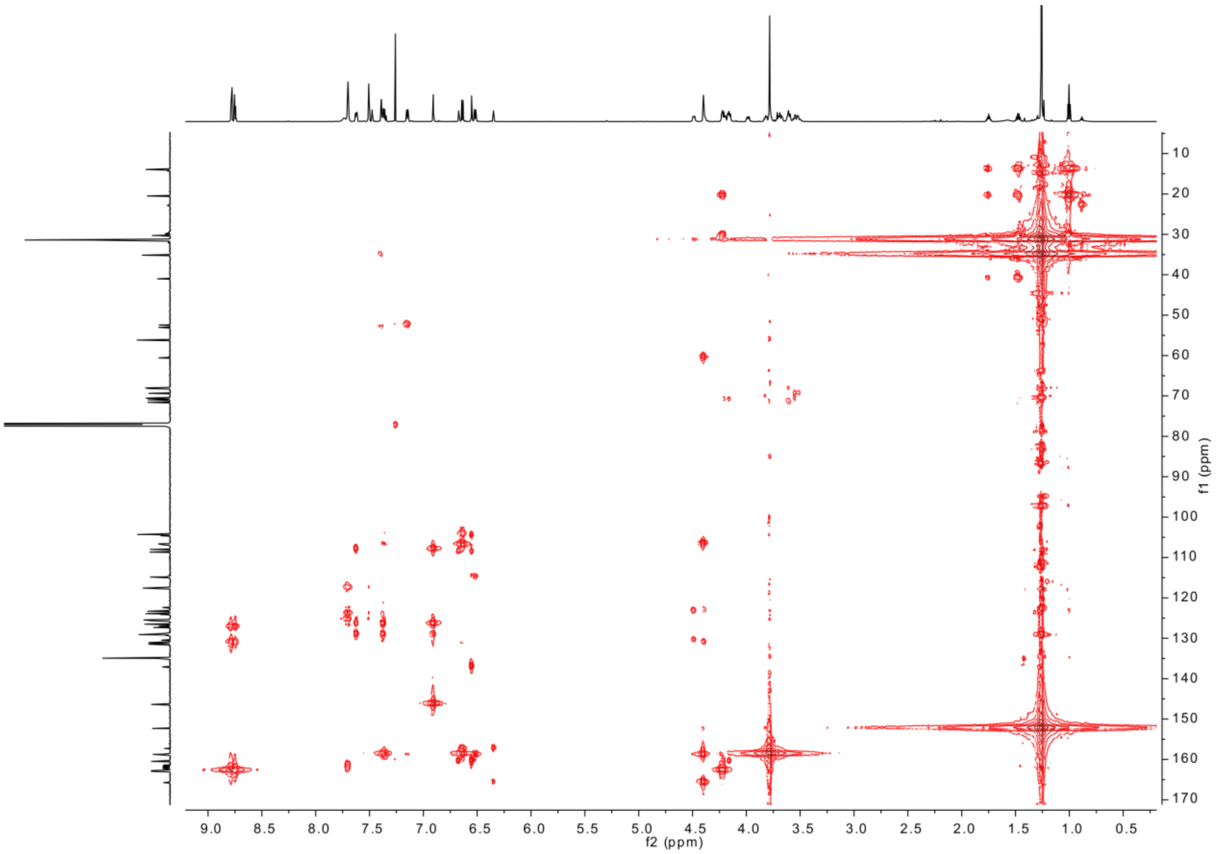

Figure S14: ^{1}H, ^{13}C HMBC NMR spectrum (700 MHz, $CDCl_3$, 298 K) of rotaxane **NDIRot**.

5.2. Tandem mass spectrometry

A Synapt G2-S HDMS (Waters Co., Milford, MA, USA) instrument with a quadrupole-time-of-flight high resolution mass detector was used to perform electrospray ionization tandem mass spectrometry. Employing collision-induced dissociation (CID) of mass-selected ions, using the following settings: flow rate 10 µL min^{-1}, capillary voltage 1.5 kV, sample cone voltage 34 V, source offset 54 V, source temperature 100 °C, desolvation temperature 20 °C, nebulizer gas 5 bar, desolvation gas flow 460 L h^{-1}. For CID, N_2 was used as the collision gas. Fragmentation experiments were conducted in the trap cell of the Synapt G2-S HDMS instrument with collision energies of 4–72 V. Data acquisition and processing was carried out using MassLynx™ (version 4.1).

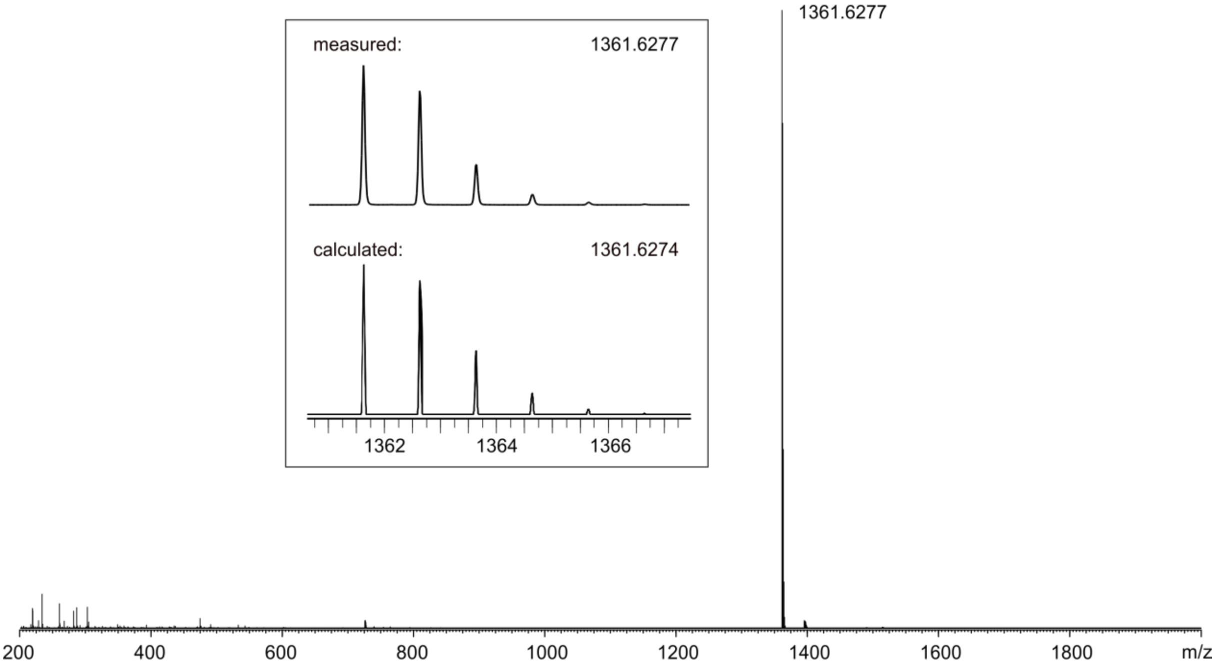

Figure S15: ESI-Q-TOF-HRMS spectrum of **NDIRot** (1 µM in CH_3CN); (inset) comparison of measured and calculated isotopic patterns.

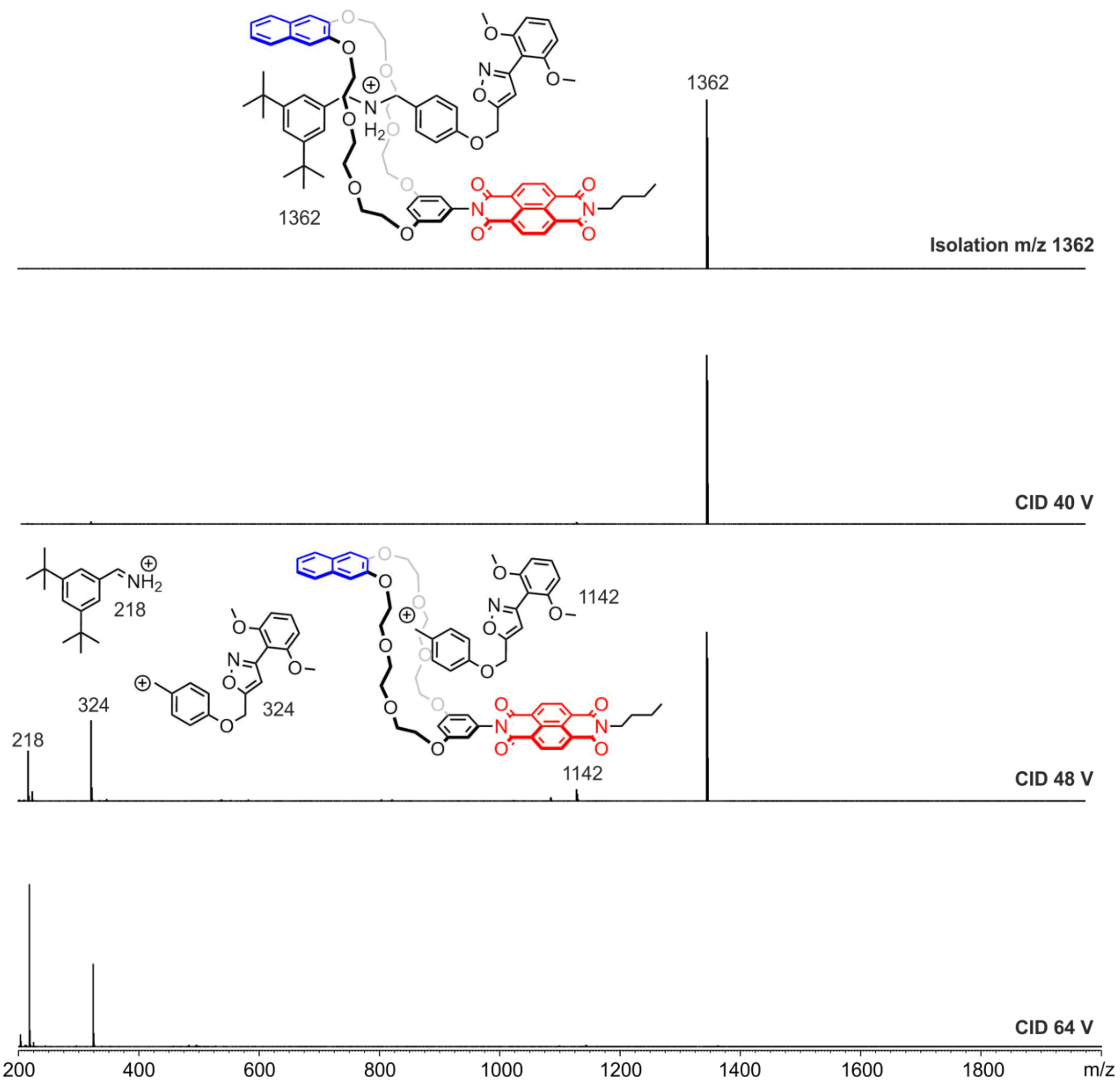

Figure S16: Collision-induced dissociation (CID) experiment with mass-selected rotaxane ions at *m/z* 1362 obtained from a CH_3CN solution (1 µM) of **NDIRot**: (top) after mass selection; (bottom) after fragmentation at different voltages. The fragment peaks could be assigned to ions formed by α-cleavage at the ammonium group of the axle. As no free axle (*m/z* 543) is observed in the fragmentation pattern, the interlocked structure of the selected rotaxane ion can be concluded.

6. Spectroelectrochemical measurements

UV–vis–NIR

The UV–vis–NIR spectroelectrochemistry measurements were performed on an Avantes spectrometer with an AvaLight-DH-S-Bal light source, an AvaSpec-ULS2048 UV/Vis detector, and an AvaSpec-NIR256-TEC NIR detector. Dry, freshly distilled and Argon purged solvents (CH_3CN and CH_2Cl_2) were used. The measurements were carried out in an optically transparent thin-layer electrochemical (OTTLE) cell (CaF_2 windows) with a platinum-mesh working electrode, a platinum-mesh counter electrode, and a silver-foil pseudoreference electrode. Voltammetric cycles between 0 and −1.2 V (vs. silver pseudoreference) were performed.

Table S3: Absorption maxima[a] (in nm) obtained by spectroelectrochemistry UV–vis–NIR measurements (0.1 M *n*-Bu_4PF_6, CH_2Cl_2/CH_3CN 1:1, 298 K).

oxidation state	NDIC7	NDIC8	NDIRot
NDI (0.0 V)	325, 342 (sh), 360, 380, 450 (sh)	324, 341 (sh), 359, 379	324, 341 (sh), 359, 379
$NDI^{\bullet-}$ (-0.6 V)	380, 402, 477, 528 (sh), 561 (sh), 592, 611, 674, 750	358, 380, 400, 476, 527 (sh), 589 (sh), 609, 685, 763	341, 359, 379, 399, 476, 527 (sh), 559 (sh), 589 (sh), 609, 689, 763
NDI^{2-} (-1.2 V)	379, 400, 422, 476, 549, 601, 675, 750	/[b]	/[b]

[a]sh = shoulder. [b]No significant band shifts and changes in absorbance were observed at more negative potential (−1.2 V vs silver wire), which can be explained by diffusion from the electrode and comproportionation of the doubly reduced species

Continuous-wave electron paramagnetic resonance spectroscopy

Continuous-wave electron paramagnetic resonance (CW-EPR) spectra at X-band frequency (ca. 9.5 GHz) were obtained with a Magnettech MS-5000 benchtop EPR spectrometer equipped with a rectangular TE 102 cavity and TC HO4 temperature controller. The measurements were carried out in synthetic quartz glass tubes. Dry, Argon purged and freshly distilled solvents were used. For the in situ preparation of the oxidized species, a three-electrode setup was employed using two Teflon-coated platinum wires (0.005″ bare, 0.008″ coated) as working and counter electrode and a Teflon-coated silver wire (0.005″ bare, 0.007″ coated) as pseudoreference electrode.

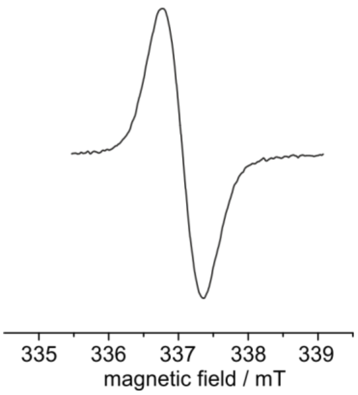

Figure S17: EPR spectroelectrochemistry measurements (0.1 M *n*-Bu_4PF_6, CH_2Cl_2/CH_3CN 1:1, 298 K) of radical species **NDIRot** (1 mM).

7. ^{1}H and ^{13}C NMR spectra

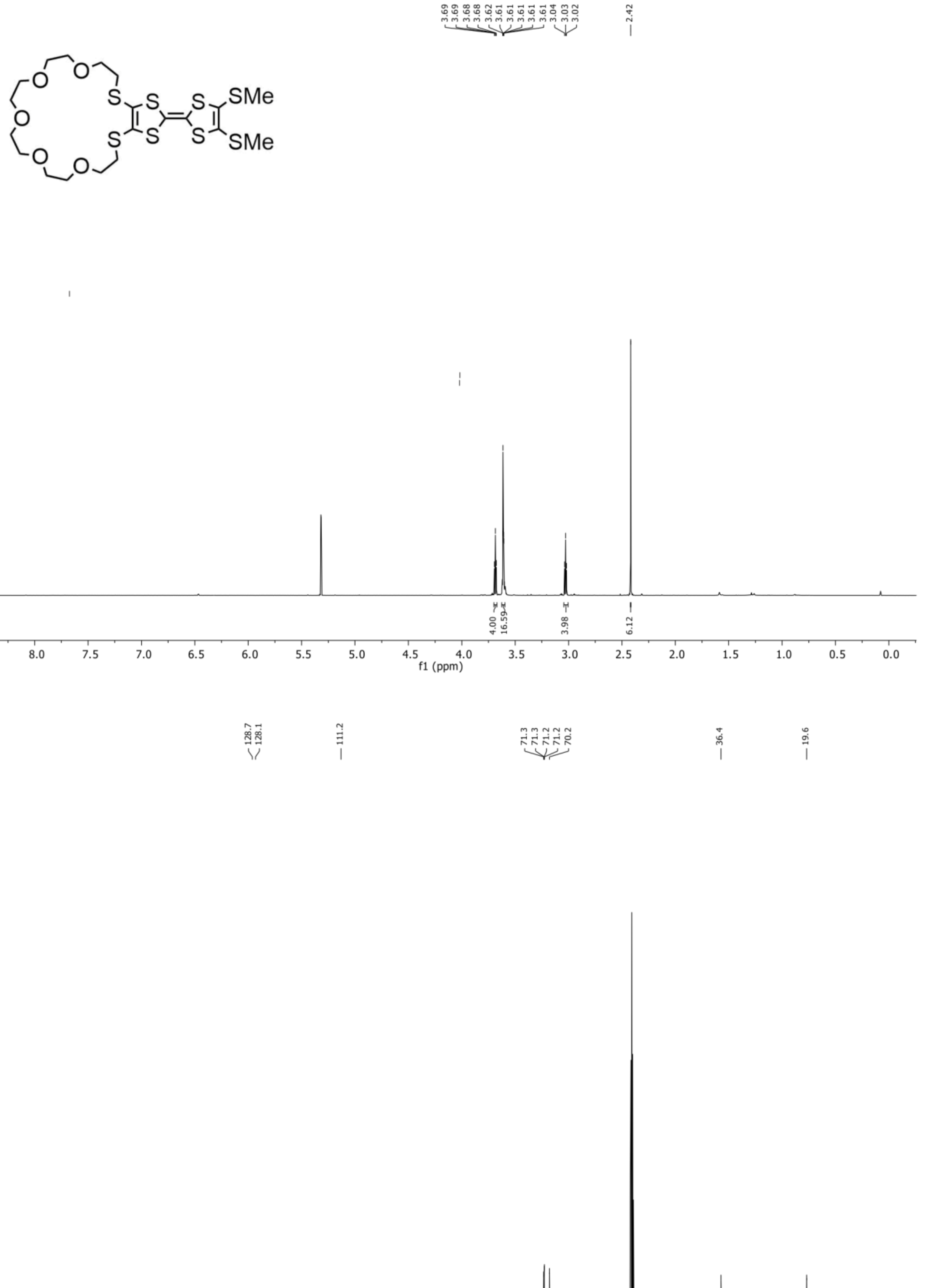

Figure S18: ^{1}H (top) and ^{13}C (bottom) NMR spectrum (700/176 MHz, CD_2Cl_2, 298 K) of **TTFC7**.

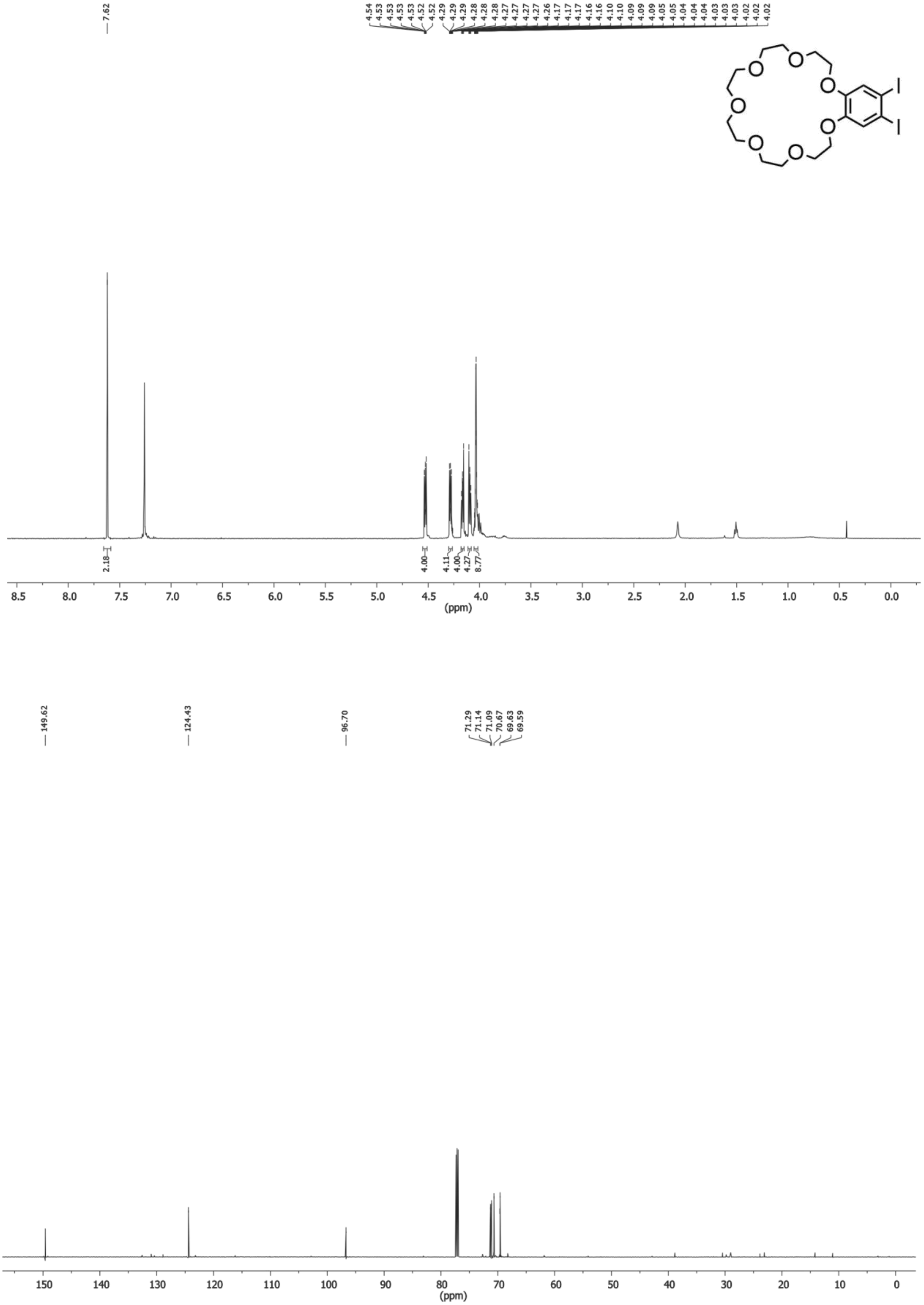

Figure S19: ^{1}H (top) and ^{13}C (bottom) NMR spectrum (500/176 MHz, $CDCl_3$, 298 K) of Diiodide **2**.

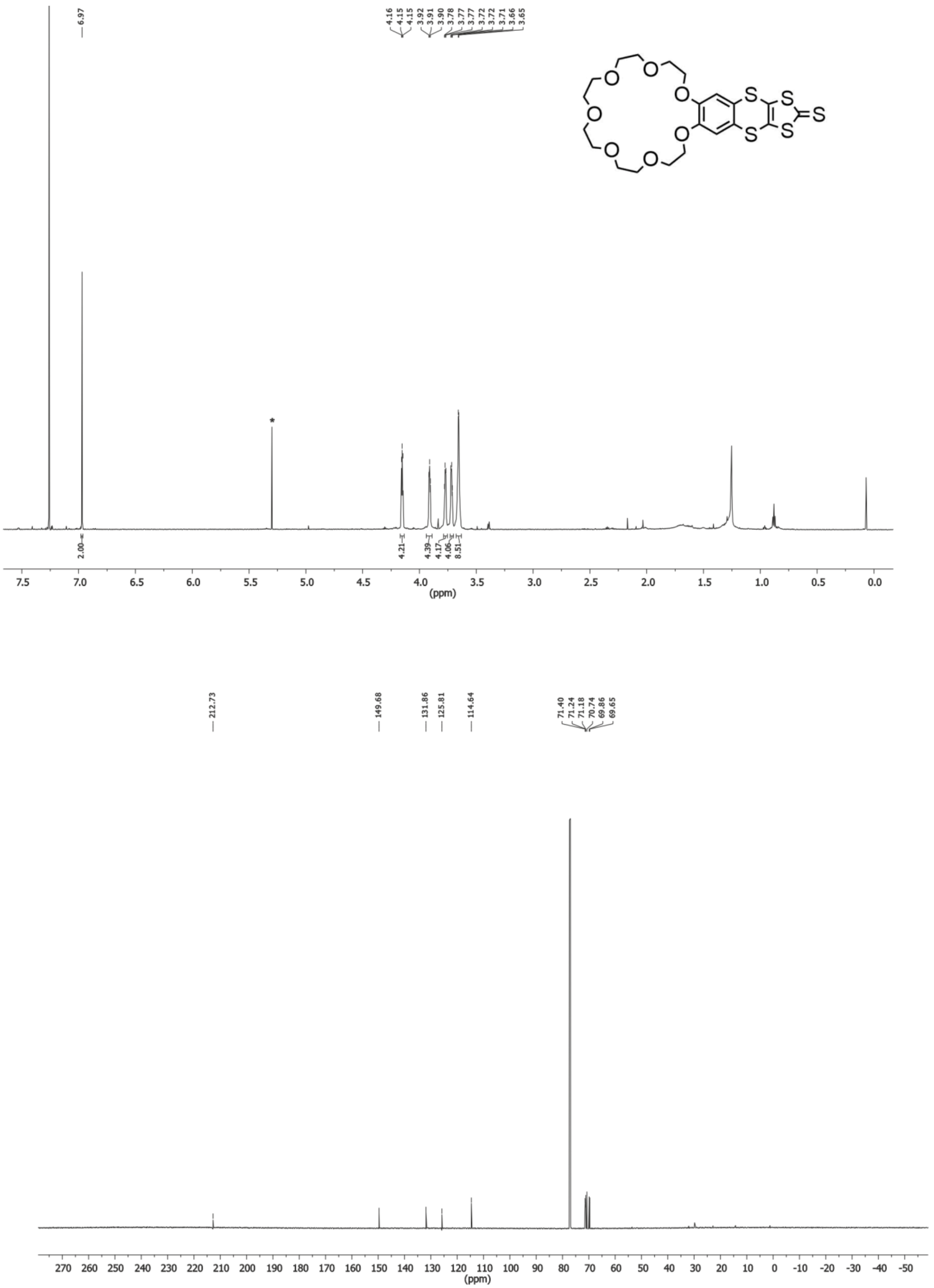

Figure S20: ^{1}H (top) and ^{13}C (bottom) NMR spectrum (500/176 MHz, $CDCl_3$, 298 K) of Thione **3**.

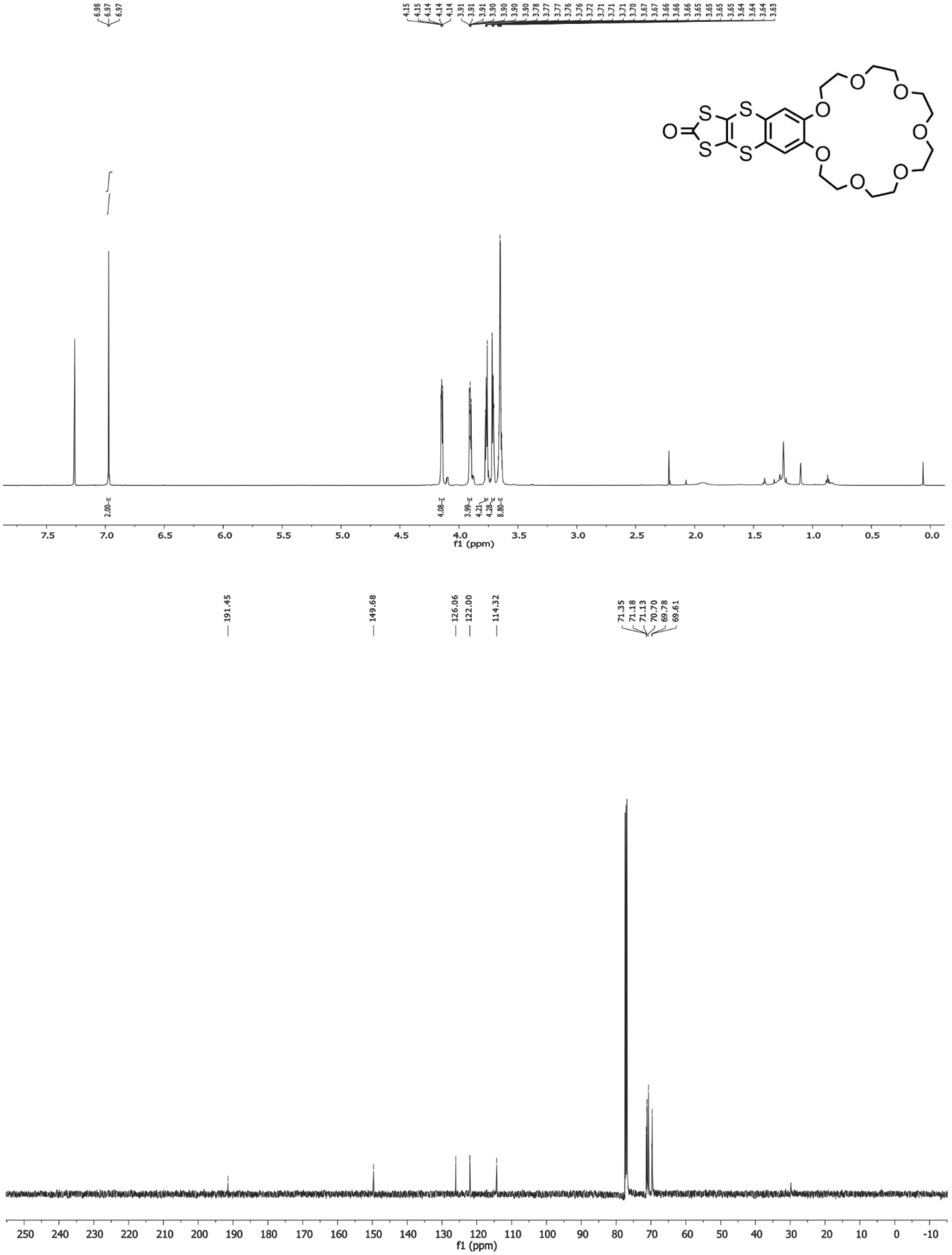

Figure S21: ^{1}H (top) and ^{13}C (bottom) NMR spectrum (500/176 MHz, $CDCl_3$, 298 K) of One **4**.

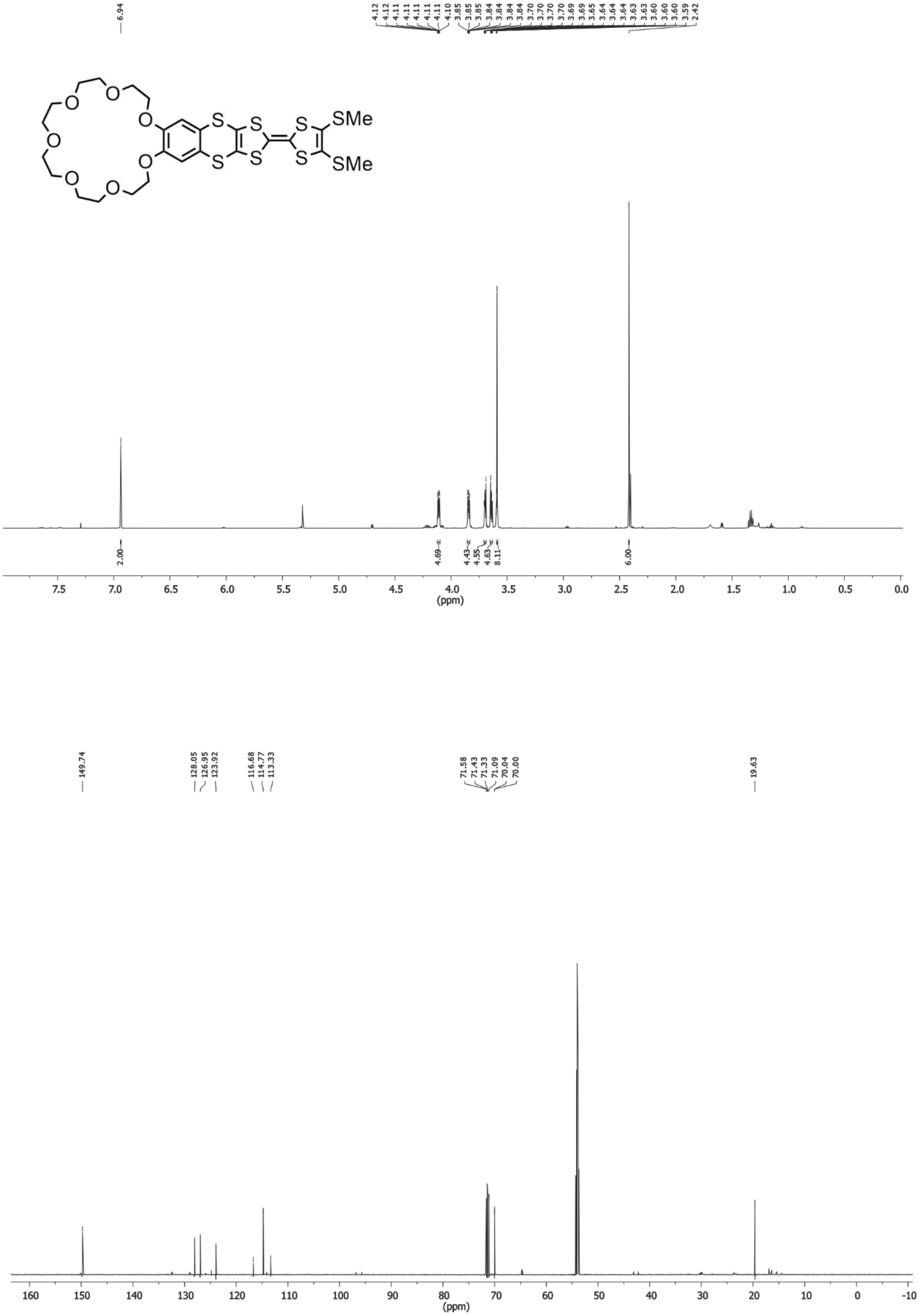

Figure S22: ^{1}H (top) and ^{13}C (bottom) NMR spectrum (700/176 MHz, CD_2Cl_2, 298 K) of **exTTFC7**.

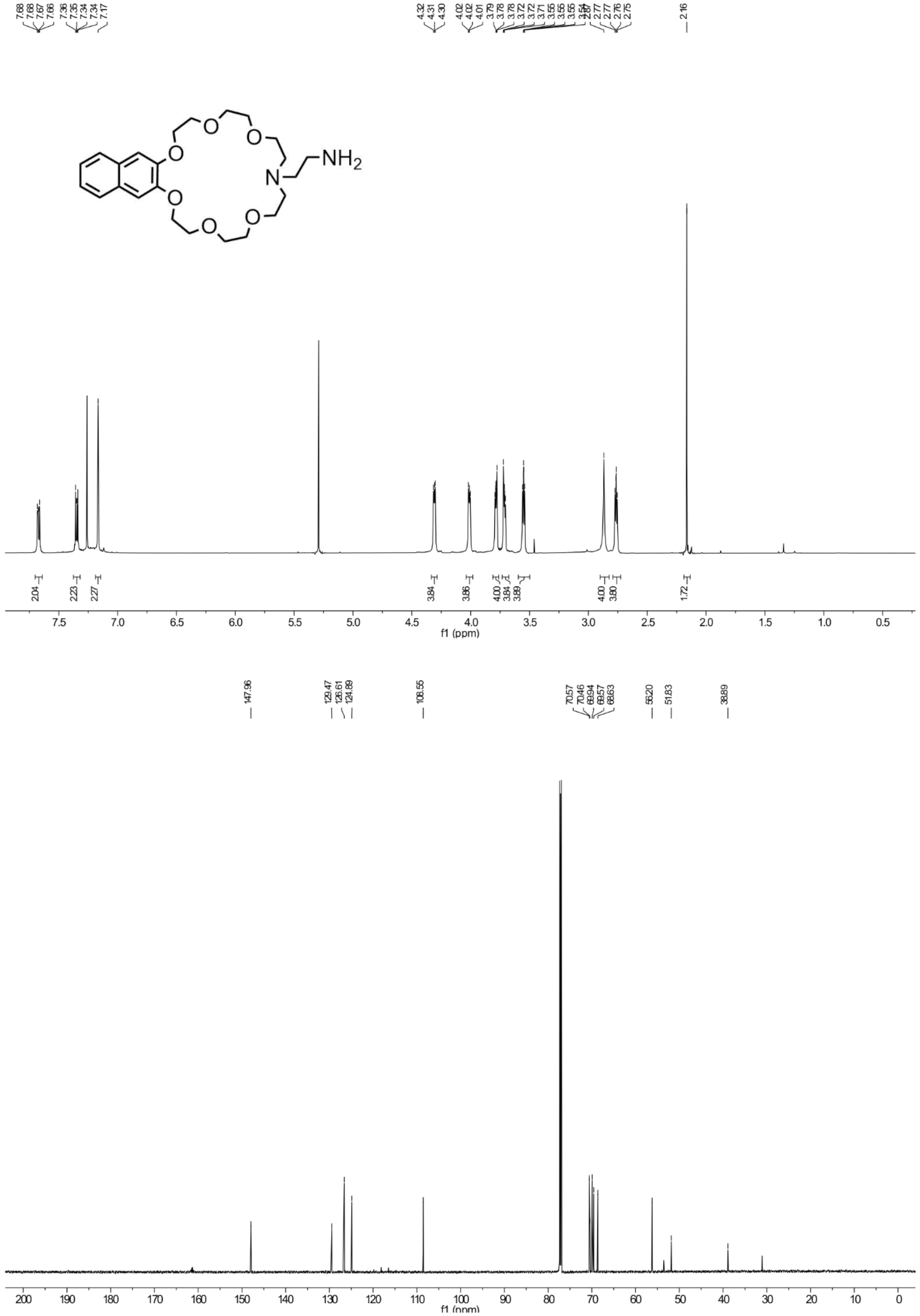

Figure S23: ^{1}H (top) and ^{13}C (bottom) NMR spectrum (500/176 MHz, $CDCl_3$ 298 K) of amine **6**.

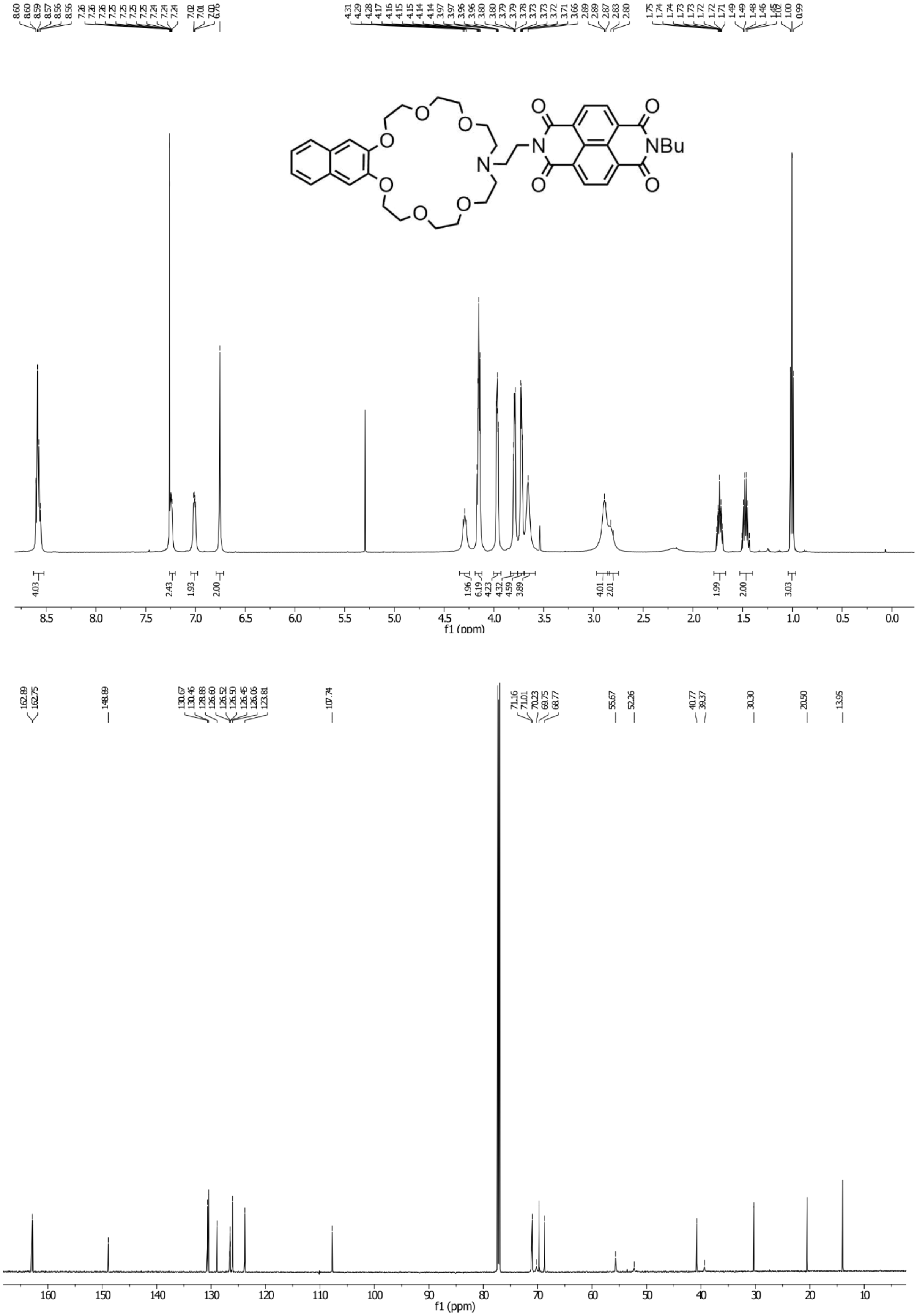

Figure S24: ^{1}H (top) and ^{13}C (bottom) NMR spectrum (500/176 MHz, $CDCl_3$, 298 K) of **NDIC7**.

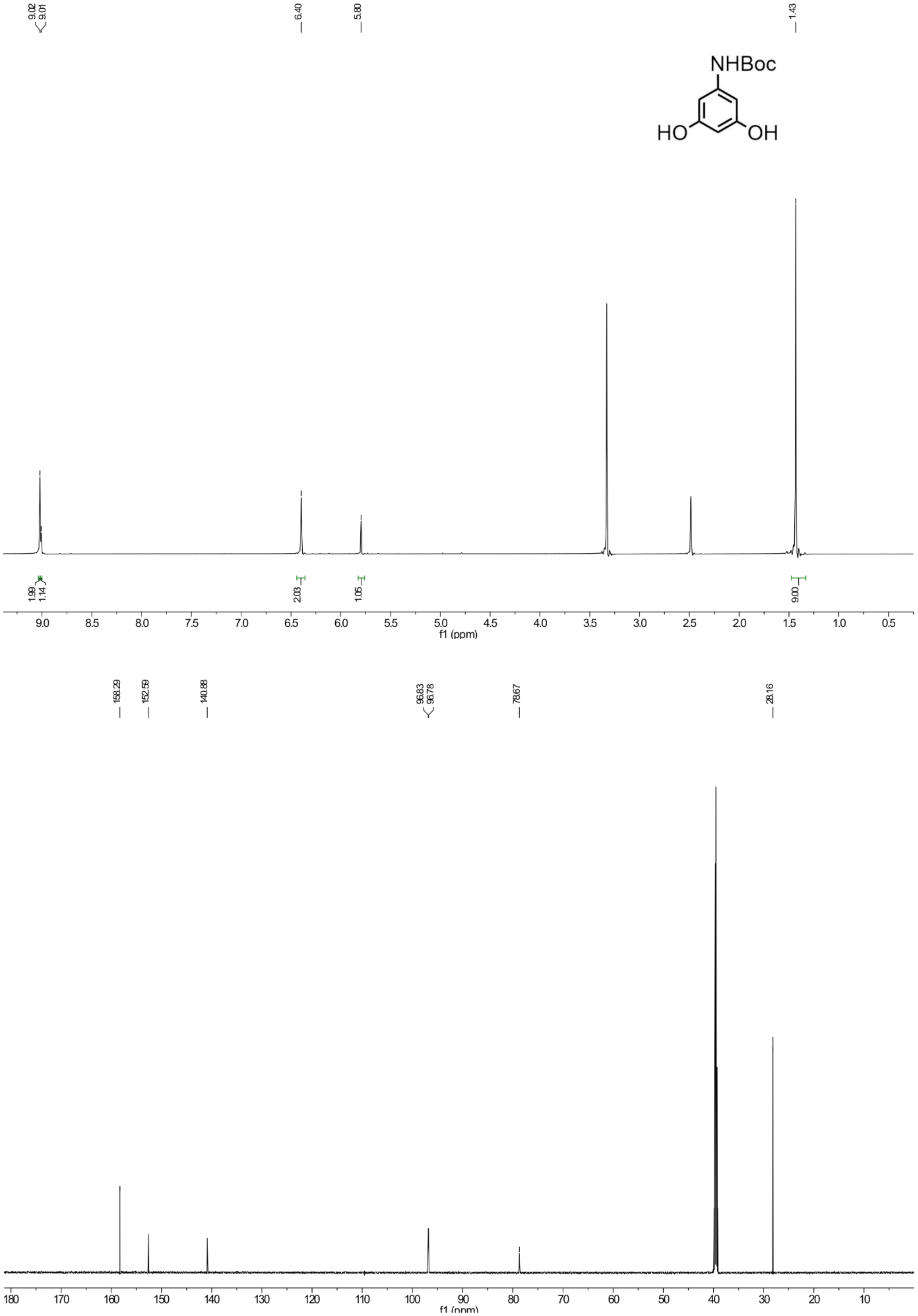

Figure S25: ^{1}H (top) and ^{13}C (bottom) NMR spectrum (700/176 MHz, $CDCl_3$, 298 K) of catechol **8**.

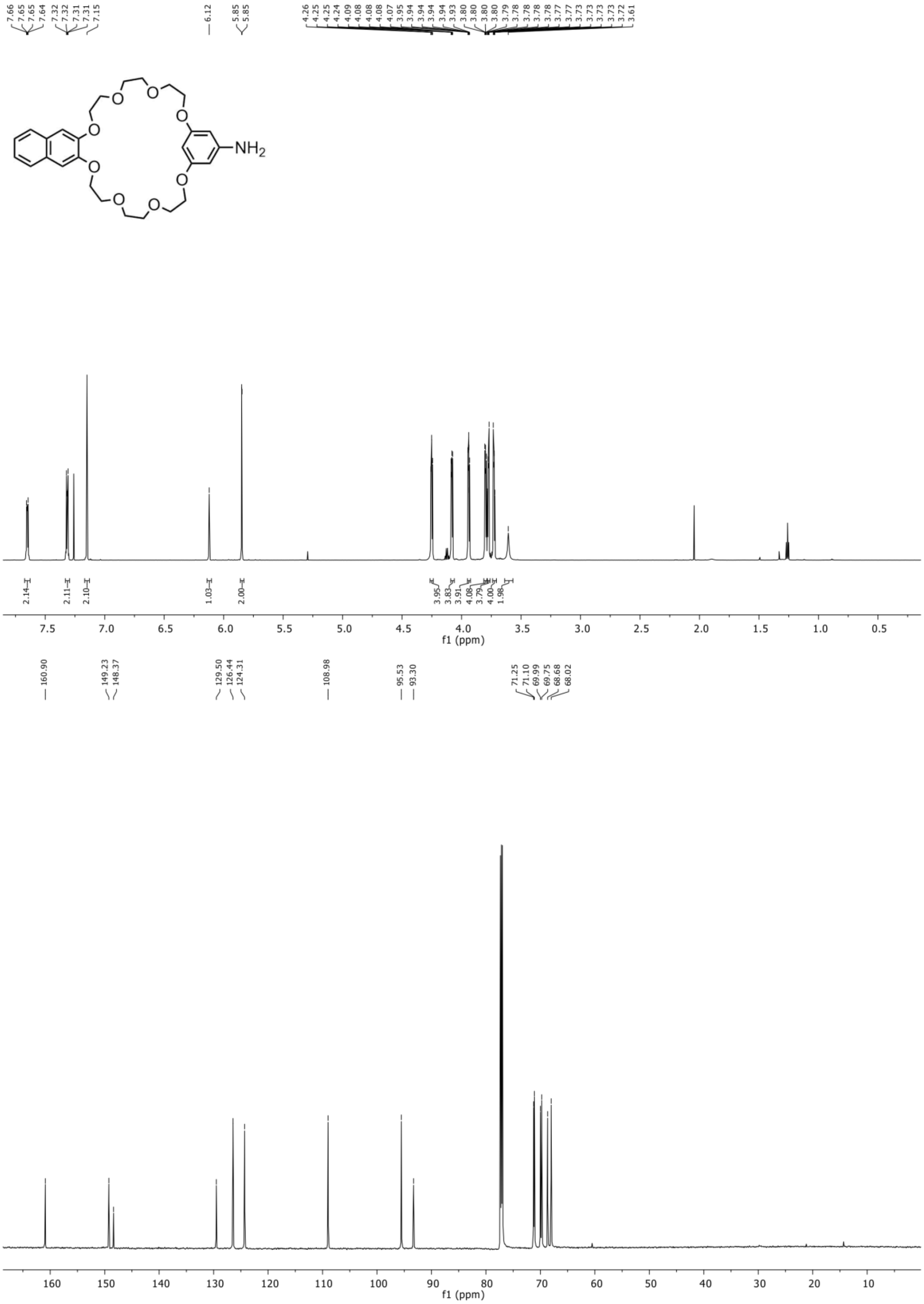

Figure S26: ^{1}H (top) and ^{13}C (bottom) NMR spectrum (700/176 MHz, $CDCl_3$, 298 K) of amine **9**.

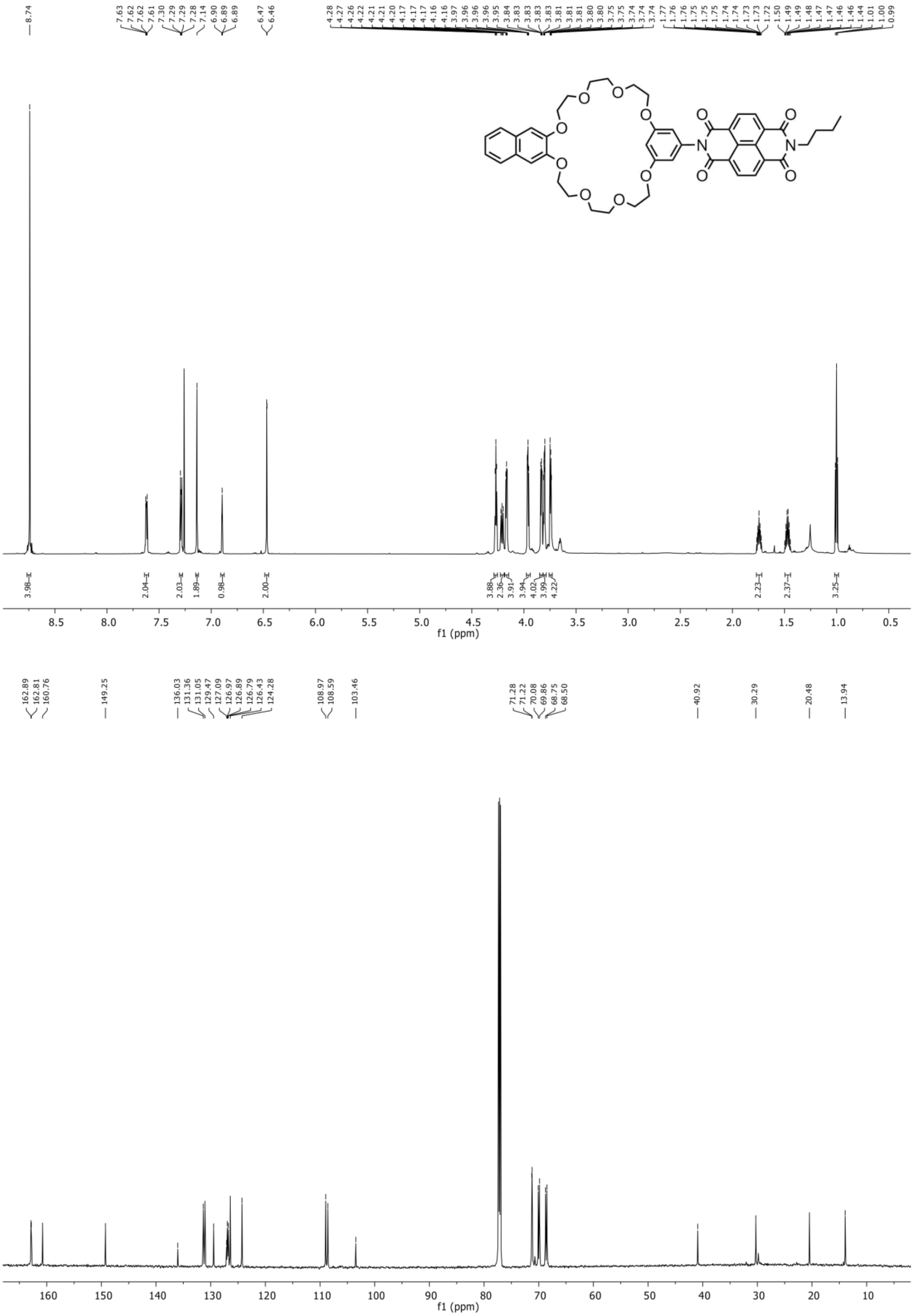

Figure S27: ^{1}H (top) and ^{13}C (bottom) NMR spectrum (700/176 MHz, $CDCl_3$, 298 K) of **NDIC8**.

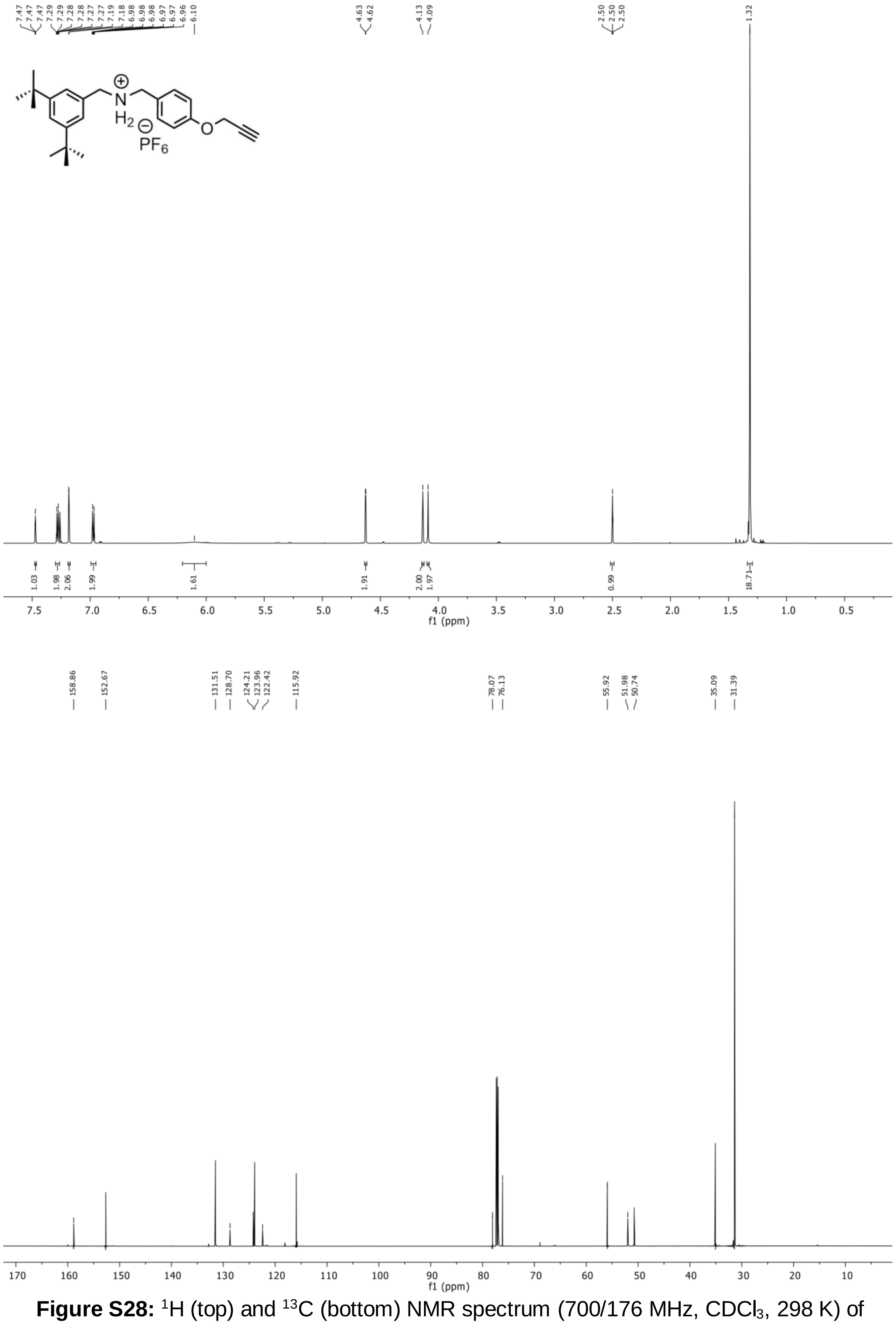

Figure S28: ^{1}H (top) and ^{13}C (bottom) NMR spectrum (700/176 MHz, $CDCl_3$, 298 K) of **A1**•PF_6.

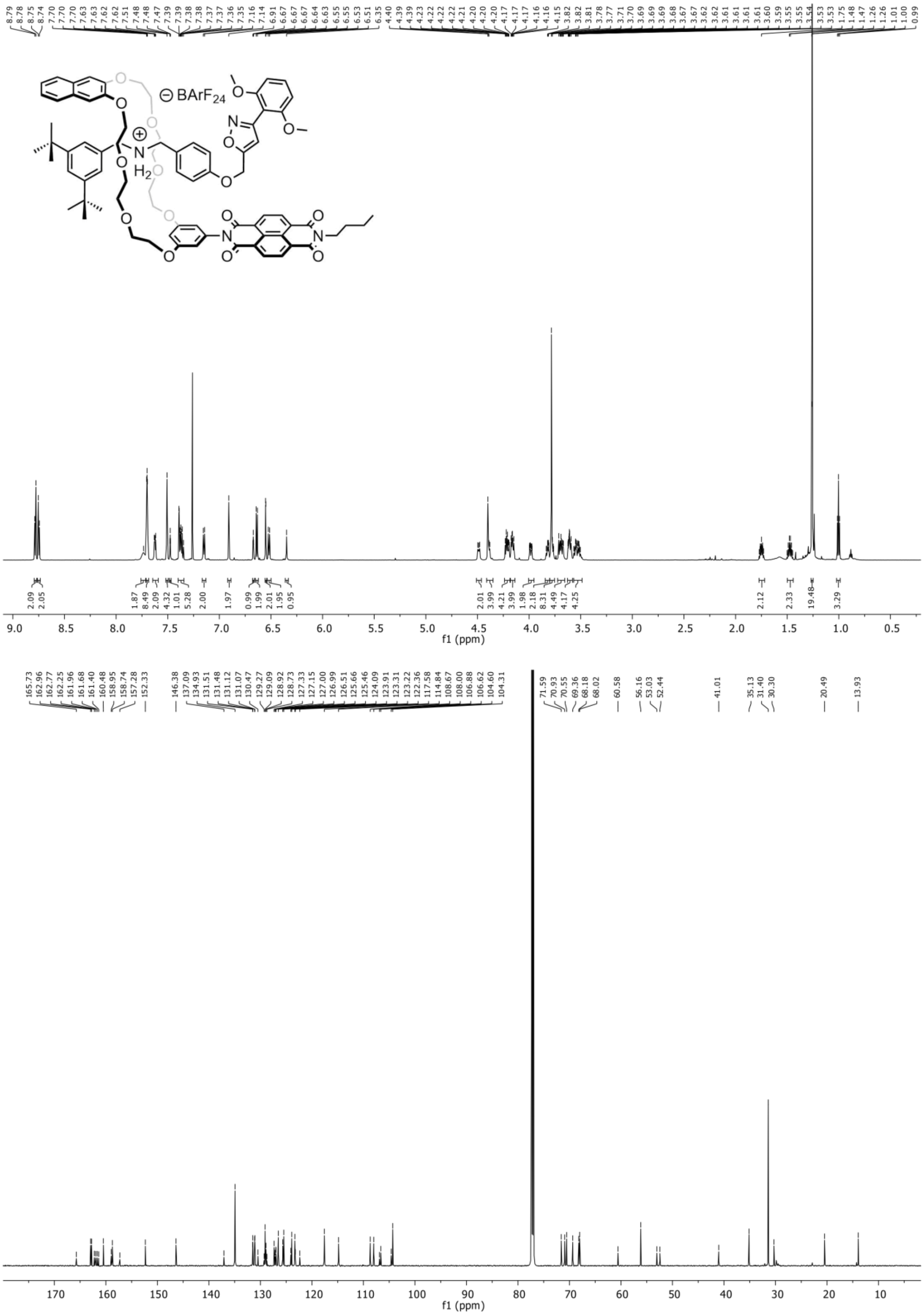

Figure S29: ^{1}H (top) and ^{13}C (bottom) NMR spectrum (700/176 MHz, $CDCl_3$, 298 K) of **NDIRot**.

8. References

1. N. Svenstrup, K. M. Rasmussen, T. K. Hansen, J. Becher, *Synthesis* **1994**, 809-812.
2. Y. Aoki, N. Umezawa, Y. Asano, K. Hatano, Y. Yano, N. Kato, T. Higuchi, *Bioorg. Med. Chem.* **2007**, *15*, 7108-7115.
3. W. Jiang, C. A. Schalley, *Beilstein J. Org. Chem.* **2010**, *6*, No. 14.
4. J. D. Badjic, C. M. Ronconi, J. F. Stoddart, V. Balzani, S. Silvi, A. Credi, *J. Am. Chem. Soc.* **2006**, *128*, 1489-1499.
5. H. Shao, T. Nguyen, N. C. Romano, D. A. Modarelli, J. R. Parquette, *J. Am. Chem. Soc.* **2009**, *131*, 16374-16376.
6. Z.-J. Zhang, H.-Y. Zhang, H. Wang, Y. Liu, *Angew. Chem. Int. Ed.* **2011**, *50*, 10834-10838.
7. C. Zhang, S. Li, J. Zhang, K. Zhu, N. Li, F. Huang, *Org. Lett.* **2007**, *9*, 5553-5556.
8. H. V. Schröder, S. Sobottka, M. Nößler, H. Hupatz, M. Gaedke, B. Sarkar, C. A. Schalley, *Chem. Sci.* **2017**, *8*, 6300-6306.
9. T. Matsumura, F. Ishiwari, Y. Koyama, T. Takata, *Org. Lett.* **2010**, *12*, 3828-3831.
10. *CrysAlisPro* (Version 1.171.39.43c.), Rigaku Oxford Diffraction, **2015**.
11. R. C. Clark, J. S. Reid, *Acta Crystallogr., Sect. A. Found. Crystallogr.* **1995**, *51*, 887-897.
12. G. M. Sheldrick, *Acta Crystallogr., Sect. A Found. Adv.* **2015**, *71*, 3-8.
13. G. M. Sheldrick, *Acta Crystallogr., Sect. C Struct Chem.* **2015**, *71*, 3-8.
14. G. M. Sheldrick, *Acta Crystallogr. , Sect. A Found. Crystallogr.* **2008**, *64*, 112-122.
15. C. B. Hübschle, G. M. Sheldrick, B. Dittrich, *J. Appl. Crystallogr.* **2011**, *44*, 1281-1284.
16. *in APEX3*, Bruker AXS Inc., Madison, Wisconsin, USA, **2015**.
17. G. M. Sheldrick, Version 2008/1, University of Göttingen, Germany, **2008**.
18. SAINT+, Version 8.27b © ed., Bruker AXS Inc., Madison, Wisconsin, USA, **1997-2012**.
19. G. M. Sheldrick, Version 2014/7 ed, University of Göttingen, Germany, **2014**.
20. J. R. Aranzaes, M.-C. Daniel, D. Astruc, *Can. J. Chem.* **2006**, *84*, 288-299.
